# Fusion transcript detection using spatial transcriptomics

**DOI:** 10.1186/s12920-020-00738-5

**Published:** 2020-08-04

**Authors:** Stefanie Friedrich, Erik L. L. Sonnhammer

**Affiliations:** Science for Life Laboratory, Department of Biochemistry and Biophysics, Stockholm University, Box 1031, 17121 Solna, Sweden

**Keywords:** Fusion transcript detection, Spatial Transcriptomics, Gene fusion, Cis-SAGE, Oncogene

## Abstract

**Background:**

Fusion transcripts are involved in tumourigenesis and play a crucial role in tumour heterogeneity, tumour evolution and cancer treatment resistance. However, fusion transcripts have not been studied at high spatial resolution in tissue sections due to the lack of full-length transcripts with spatial information. New high-throughput technologies like spatial transcriptomics measure the transcriptome of tissue sections on almost single-cell level. While this technique does not allow for direct detection of fusion transcripts, we show that they can be inferred using the relative poly(A) tail abundance of the involved parental genes.

**Method:**

We present a new method STfusion, which uses spatial transcriptomics to infer the presence and absence of poly(A) tails. A fusion transcript lacks a poly(A) tail for the 5′ gene and has an elevated number of poly(A) tails for the 3′ gene. Its expression level is defined by the upstream promoter of the 5′ gene. STfusion measures the difference between the observed and expected number of poly(A) tails with a novel C-score.

**Results:**

We verified the STfusion ability to predict fusion transcripts on HeLa cells with known fusions. STfusion and C-score applied to clinical prostate cancer data revealed the spatial distribution of the cis-SAGe *SLC45A3-ELK4* in 12 tissue sections with almost single-cell resolution. The cis-SAGe occurred in disease areas, e.g. inflamed, prostatic intraepithelial neoplastic, or cancerous areas, and occasionally in normal glands.

**Conclusions:**

STfusion detects fusion transcripts in cancer cell line and clinical tissue data, and distinguishes chimeric transcripts from chimeras caused by trans-splicing events. With STfusion and the use of C-scores, fusion transcripts can be spatially localised in clinical tissue sections on almost single cell level.

## Background

A fusion transcript is a merging of different fragment transcripts. This molecule can be translated into a chimeric protein that possesses either a new or adapted function. Many fusion transcripts are classified as oncogenes that have the potential to cause cancer but also contribute to tumourigenesis as driver mutations. Mitelman et al. [1] estimated that 20% of cancer morbidity is caused by such fusions. Currently, 21,477 fusion transcripts have been identified; almost all can be found in neoplastic cells [2]. The majority of chimeric transcripts were detected in recent years by deep-sequencing technologies and the application of bioinformatics tools [2]. The occurrence of fusion transcripts is used as a cancer biomarker [3] as well as a cancer treatment target [4].

Fusion transcripts, also termed chimeric transcripts or chimeric RNA, are usually detected at the RNA level. If its underlying cause is known, it is further specified as either a genetic (i.e., gene fusion) or transcription-induced chimera. Gene fusions are caused by a genetic mutation – deletion, inversion or translocation event – of the DNA sequence from both parental genes. The mutated parental gene DNA sequences are translated into a hybrid messenger RNA (mRNA). Whereas transcription-induced chimeras are caused by an abnormal mechanism either cis-splicing or trans-splicing [5]. The parental gene sequences remain intact.

Cis-splicing fusion transcripts are termed cis-splicing of adjacent genes (cis-SAGe) or read-through transcripts. Cis-SAGe result from neglected gene boundaries; instead the DNA sequences of two adjacent genes are read and transcribed into a hybrid mRNA transcript. Cis-SAGe were not exclusively identified in cancer cells. Despite intense research on clinical tissues and cancer cell lines that harbour a cis-SAGe, there is no convincing genetic mutation behind their production and thus a molecular mechanism is suspected. Chimeric transcription of cis-SAGe requires two genes on the same strand within 30 kilobase pairs (kbp) [6]. Chwalenia et al. [7] summarised features of cis-splicing chimeras: (i) active transcription of the 5′ gene, (ii) multi exonic neighbouring parental genes, (iii) absence of interstitial DNA deletion, (iv) presence of transcripts between the two neighbouring genes, (v) presence of CTCF binding sites between parental genes and (vi) induction by CTCF knockdown. They further suggest that cis-SAGe are an additional element of biological processes that increases the diversity of gene products. This supposition is consistent with the fact that cis-SAGe are also found in healthy tissue samples. Different cis-SAGe fusion variants, which are characterised by the different fusion points of the parental genes, are observed. Dominant among the so far detected cis-SAGe is the fusion point at the second exon of the 3′ gene [7].

Transcription induced fusion transcripts that result from trans-splicing are hybrids of two mRNA transcripts of separately transcribed genes. These molecules are rare, but if they occur, they can contribute to neoplastic transformation due to their pro-proliferative effects [8].

The poly(A) tail is a 100–250 bp sequence of adenine nucleotides. The poly(A) tail is not part of the DNA sequence; it is attached to the 3′ gene post-transcriptionally. A poly(A) signal at the 3′ untranslated region (UTR) of the DNA sequence defines the point of the poly(A) tail synthesis. Poly(A) polymerases, which are RNA polymerases, encode and attach the poly(A) tail to the pre-mRNA. Cleavage and polyadenylation specificity factor (CPSF) is one representative protein that recognises the poly(A) signal in the pre-mRNA sequence (in eukaryotes, often AAUAAA). Although the function of the poly(A) tail is not completely known, it increases mRNA stability during export from the nucleus to the ribosome, protects the mRNA from degradation and regulates its half-life. The poly(A) tail shortens with mRNA age. Further, the poly(A) tail, together with the counterpart at the 5′ end (the 5′ cap), initiates protein translation [9]. Recent studies by Park et al. [10] investigated its regulatory role in the somatic cell cycle. Cell cycle dysregulation is a hallmark of cancer.

Chimeric RNA can be detected experimentally (e.g. fluorescence in situ hybridisation [FISH] or Southern blot) or computationally. The latter is based on RNA-sequencing (RNA-seq) data and the application of software tools that identify the chimeric RNA. The tools search for encompassing reads, i.e., read pairs with each read aligned to one of the parental genes, and spanning reads, i.e., partially aligned reads that span the fusion point. Sensitivity and specificity depend on the tool as well as read length, read quality score and the number of reads that support each fusion transcript [11, 12]. The results are often compared to known fusion transcripts and then categorised into potential gene fusions, cis-SAGe or trans-splicing fusion transcripts. This search strategy, however, provides no information about the transcription direction and is limited in terms of spatial information.

In this study, we present a novel method to detect fused mRNA molecules using the poly(A) tail presence at the 3′ gene and its absence at the 5′ gene. Applying this method, and the C-score, to clinical tissue sections analysed with spatial transcriptomics allowed the detection of the cis-SAGe *SLC45A3-ELK4* on almost single cell level. The cis-SAGe clearly overlapped with disease areas within the tissue samples. Further, we emphasise that increased cis-SAGe correlates with elevated levels of transcriptional stress.

## Methods

### Fusion transcript detection using STfusion and poly(A) tail presence

During the correct transcription of two adjacent genes, the poly(A) tail is attached to each of the genes. The poly(A) tail serves as a proxy for the transcription level of each gene.

**Fig. 1:**
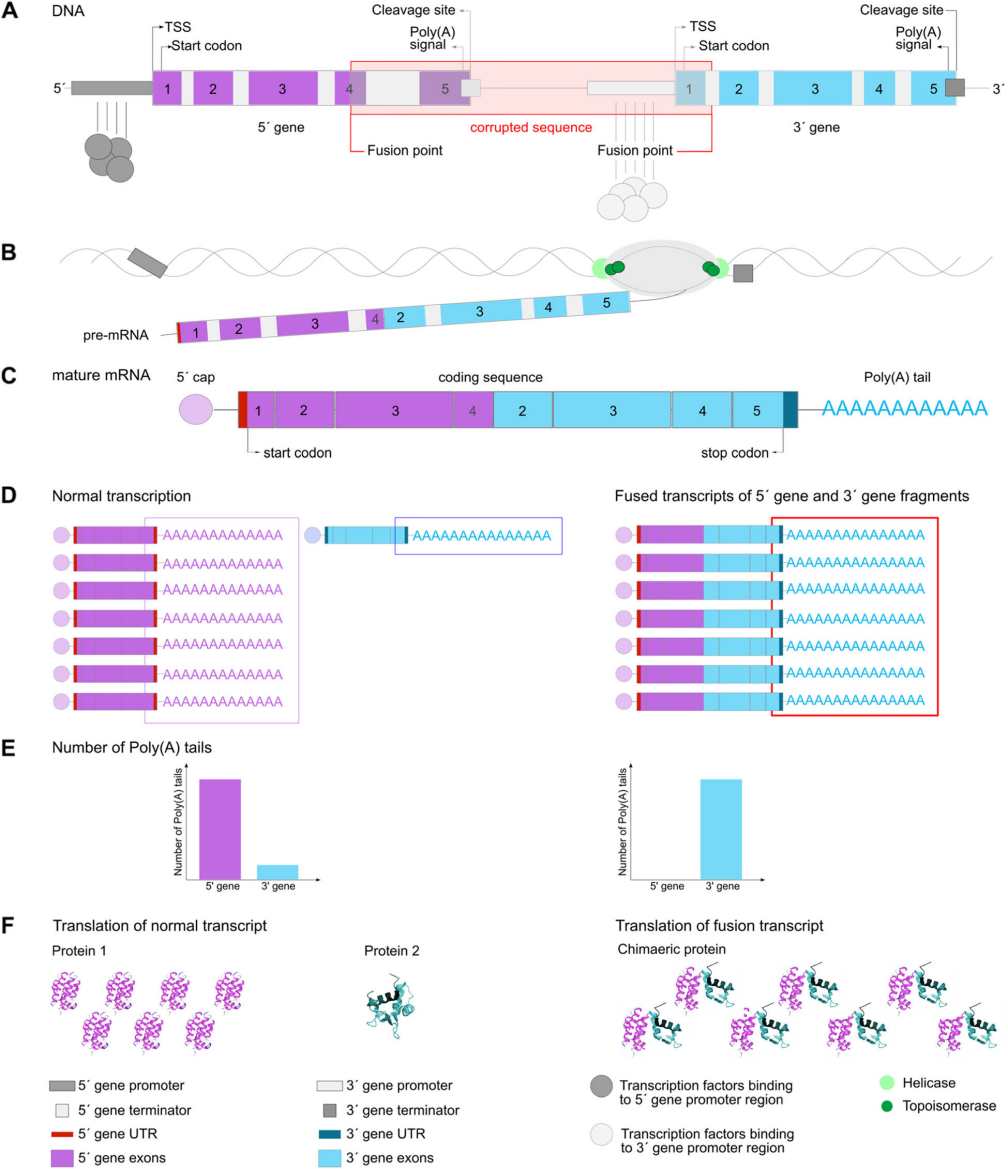
Expression of a fusion transcript. **a** Gene structure of the parental genes. The corrupted region between both genes leads to the fusion transcript. The fusion point is set to exon 4 of the 5′ gene and the intronic region between exons 1 and 2 for the 3′ gene. **b** Transcription of the fusion transcript is shown. Only fragments of the 5′ gene and fragments of the 3′ gene are transcribed. During transcription, helicase divides the two strands to allow RNA polymerase to act; both strands are subsequently combined. Topoisomerase avoids rotation and tension of the DNA strands during de-spiralisation. **c** Polyadenylation during post-transcriptional modification of the chimeric transcript and splicing to a mature mRNA. **d** Expression levels are defined by the promoter in the 5′ UTR of each gene. Normal transcription of the adjacent genes involves a different number of poly(A) tails on the parental genes (left). Transcription of the fusion transcript, however, results in an expression level based on the 5′ gene promoter and an elevated number of poly(A) tails attached to the 3′ UTR (right). **e** The difference in the number of poly(A) tails between normal, not fused (left barplot) and fused (right barplot) transcripts of the parental genes leads to the detection of the fusion transcript. If fused, the number of poly(A) tails on the 5′ gene becomes 0 and on the 3′ gene equal to the number of poly(A) tails on the normal 5′ gene transcript. **f** mRNA is translated and then folded into a protein. In case of a translated fusion transcript the folded protein is chimeric, i.e., a combination or section of fragments from the parental genes

In the case of a fusion transcript caused by a cis-SAGe mechanism or chromosomal rearrangement, however, the poly(A) tail is absent from the 5′ gene. In this case, the 5′ gene expression level defines the fusion transcript expression by the promoter of the 5′ UTR of the 5′ gene. The poly(A) tail is attached to the 3′ end of the parental 3′ gene. In the proposed method, the number of sequenced poly(A) tails that can be mapped to the 3′ gene, and the absence of poly(A) tails at the 5′ gene, is used to indicate a fusion transcript. The number of poly(A) tails aligned to the 3′ gene mirrors the expression level of the fusion transcript (Fig. 1, Table S12).

### STfusion verification in HeLa cells

In order to verify STfusion, sequenced mRNA from HeLa cancer cells were analysed. The occurrence of poly(A) tails aligned to the parental genes of experimentally confirmed fusion transcripts was tracked (Tables S1-S11).

The sequenced mRNA data produced by TAIL-seq [13], and the results published in the same paper, were used. This tool sequenced the end of the mRNA and thus includes the poly(A) tail sequence. Chang et al. [13] applied TAIL-seq to HeLa and found 4000 genes have a poly(A) tail.

Additionally, for parental genes of the known HeLa fusion transcripts with no poly(A) tails, according to the results by Chang et al. [13], the raw RNA-seq data from the same publication were aligned and analysed in-depth. We were only interested in reads that contain a poly(A) tail sequence, which is the file that contains the second read (read 2, read length 230 bp). First, the potential poly(A) tail sequence nucleotides were removed. Second, the reads were further trimmed by 64 nucleotides (nt) to remove additional adapter sequences. The trimming was performed with Cutadapt [14]. The resulting reads were aligned with Tophat2 [15] and STAR [16] to the human assembly GRCh38 Ensembl (release 84) [17]. Uniquely mapped reads (Tophat2 mapping quality ≥10, STAR mapping quality = 255) were kept. Finally, reads that were aligned to a reference sequence with multiple adenine or thymine nucleotides in the direct vicinity were removed, because the eliminated sequence assumed to be a poly(A) tail is part of the genome. All reads that mapped to the 3′ UTR of the parental genes were considered (Table S13).

### STfusion applied to clinical tissue samples

#### Spatial transcriptomics data, transcriptomic factors and activity maps

Spatial transcriptomics [18] (The Spatial Transcriptomics method, Suppl.), a novel technology, allows one to obtain expression levels throughout tissue samples while maintaining spatial information. Spatial transcriptomics opens new possibilities for the investigation of altered expression levels, especially under modified conditions (e.g., cancerous cells within tissue samples). In a recent publication, Berglund et al. [19] showed that the cells in the centre, periphery and vicinity of prostate cancer areas develop a unique expression pattern that is clearly differentiated from areas with healthy cells of a similar type. Thus, this technique can provide insights into cancer progression.

Spatial transcriptomics produces very rich expression levels data throughout a tissue sample. In order to identify hidden patterns of gene expressions that characterise cell types, spatial transcriptomics decomposition (STD) was developed by Maaskola et al. [20]. This method calculates expected gene expression (read counts) as the matrix product of observed gene expression and spatial activity matrices. The revealed unique expression profiles, i.e. transcriptomic factors, across tissue sections, represent different cell types, microenvironments or tissue components. For each identified expression profile, the method provides a spatial activity map that represents where the transcriptomic factor is active in the tissue sample. For example, the transcriptomic factor that represents “cancerous epithelial” cells exhibits a unique expression pattern that reveals genes strongly or differentially expressed compared to another transcriptomic factor. The activity map for the transcriptomic factor “cancerous epithelial” shows where the expression pattern is active within a tissue sample.

Berglund et al. [19] applied spatial transcriptomics to 12 tissue sections obtained from a patient diagnosed with prostate cancer. The spatial transcriptomics data comprised the expression levels of 5053 protein-coding genes in 1007 spots in each of the tissue sections. Further, the 12 tissue sections were analysed with STD in different joint approaches: (i) samples 1.2, 2.4 and 3.3 joined and (ii) the 12 tissue sections joined. The spatial transcriptomics data, STD transcriptomic factors and activity maps were used in this paper to localise the cis-SAGe *SLC45A3-ELK4*, link it to disease areas, calculate differentially expressed genes and perform pathway annotation.

#### Fusion transcript localisation using STfusion and C-scores

In spatial transcriptomics, the poly(A) tail of a transcript is captured and measured as a proxy for the expression level of a gene in a tissue sample on an almost single-cell level. However, for a gene that is abnormally transcribed, as it is the case for a fusion transcript, the amount of poly(A) tails provides shifted results. This deviation is measured.

For each parental gene, the ratio (R) of the gene expression in each spot divided by the sample mean expression was calculated (eq 1). The C-score of a spot is the maximum value of both ratios and presents the presence or absence of the fusion transcript (eq 2). In the case of absence, the C-score level mirrors the 5′ gene expression level. In the case of a fusion transcript, the C-score level mirrors the fusion transcript expression level.

1 $$ {R}_{5\hbox{'}}=\frac{5\hbox{'} gene}{5\hbox{'} gene s}\kern0.5em ;\kern1em {R}_{3\hbox{'}}=\frac{3\hbox{'} gene}{3\hbox{'} gene s} $$

2 $$ {\displaystyle \begin{array}{l} if\kern0.5em {R}_{5\hbox{'}}\ge \kern0.5em {R}_{3\hbox{'}}\kern1em C- score=-{R}_{5\hbox{'}}\\ {} otherwise\kern1.62em C- score=+{R}_{3\hbox{'}}\end{array}} $$

The proposed poly(A) tail detection method, STfusion using C-scores, was applied to the 12 clinical tissue samples analysed with spatial transcriptomics. The level of the C-score mirrors an abnormally high amount of poly(A) tails on the 3′ gene *ELK4* and predicts the cis-SAGe *SLC45A3-ELK4*.

To avoid divisions of 0, a pseudo-count of 1 can be added to both dividend and divisor of the ratios R5′ and R3´. The spatial distribution of the C-scores then changes slightly which can be circumvented using a threshold (C-score with pseudo count, Suppl., Tables S20, Figs. S4, S6 and S7).

#### Differentially expressed genes and pathway annotation

Spots with fusion transcript presence and absence were compared to investigate differentially expressed and co-expressed genes and activated pathways. Spots were only chosen according to their C-score, thus the likelihood and expression level of the cis-SAGe, and regardless of an annotation as stroma or epithelial.

To assign a spot to the group ‘occurrence’ or ‘absence’, C-score thresholds were applied:

- (i) Absence *C*-*score* < 0
- (ii) Occurrence 0 < *C*-*score*

The spatial transcriptomics data with read counts for the 5053 protein-coding genes across the spots, were checked for quality. Spots with a log-library size smaller than three median absolute deviations below the median log-library size were removed. Low-abundance genes with a read count of zero or close to zero among the spots were removed. The resulting data set was normalised per tissue sample using the R package “scran” [21]. The optimal pool size was calculated with the R package “scater” [22]. Genes with a very low standard deviation (sd) for the normalised expression levels among the chosen spots (sd < 10% of the expression mean) were removed.

The fold change per gene was calculated as gene expression mean of spots with C-scores > *0* (occurrence) divided by gene expression mean of spots with C-scores < 0 (absence). Differentially expressed genes were calculated with a two-sample t-test (confidence level 0.95) [23]. *P*-values were corrected for multiple testing with the Benjamini-Hochberg procedure [24]. Significantly differentially expressed genes (false discovery rate [FDR], q-value < 0.1) were submitted to PathwAX [25] on the Kyoto Encyclopaedia of Genes and Genomes (KEGG) database [26].

#### Detection of fusion transcript candidates

To identify a fusion transcript candidate caused by a cis-SAGe mechanism or a structural mutation (gene fusion) among random gene pair combinations, the diversity index *D* can be used; a higher value can indicate a fusion transcript. For each gene pair *i*, the diversity index is calculated as (eq. 3), where *N*_*i*_ is the number of C-scores ≠ 0, and *U*_*i*_ is the number of unique C-scores ≠ 0, in both cases rounded to one decimal point:

3 $$ {D}_1={N}_1\times {U}_1 $$

To increase the amount of data, the sample-wise calculated C-scores were concatenated for the 12 tissue samples to calculate *D*_*i*_.

### Fusion transcript detection in bulk sequenced RNA from prostate cancer tissue samples

Bulk-sequenced mRNA from each of the 12 tissue sections were used to confirm the cis-SAGe *SLC45A3-ELK4*. The sequenced reads were aligned using two aligners to increase the possibility of identifying the cis-SAGe. The alignments of fastq reads were performed using Tophat2 (b2-sensitive and otherwise default parameters) [15] and STAR [16], both alignments against the human assembly GRCh38 Ensembl (release 84) [17]. Conversion from sam to bam format and indexing was done using Samtools [27].

Fusioncatcher [28] using Blat [29], Star and Bowtie2 [30] was applied to the aligned RNA-seq data to confirm the cis-SAGe. Additionally, the alignments were searched for encompassing reads, i.e., read pairs with each of the reads mapped to one of the parental genes, and for spanning reads that covered the fusion points identified with Fusioncatcher. The search was performed using Samtools (Tables S16 and S17).

## Results

### STfusion verification in HeLa cells

To verify STfusion accuracy, we applied it to HeLa cancer cells. P. Wu et al. [31] experimentally identified nine chimeric RNAs in HeLa cells. Further detected in Hela cancer cells were the cis-SAGe *SLC45A3-ELK4* by Zhang et al. [32] and the trans-splicing fusion event *VMP1-RPS6KB1* by L. Wu et al. [33]. Of these 11 fusion transcripts, the number of poly(A) tails per parental gene were considered. If the concept is correct and a chimeric transcript caused by a cis-SAGe mechanism or a chromosomal rearrangement is transcribed, the 5´ genes should not have a poly(A) tail, but the 3′ genes will have one.

STfusion verification was performed using the sequenced mRNA and the number of poly(A) tails produced by TAIL-seq, and the published results of counted poly(A) tails per gene [13].

#### STfusion verification for gene fusions and cis-SAGe

*LHX6-NDUFA8*, *SLC2A11-MIF*, and *SLC45A3-ELK4* are confirmed cis-SAGe events in HeLa [31, 32]. The parental genes *SLC45A3* and *ELK4* were not listed as having poly(A) tails in Chang et al. [13]. With an in-depth search in the sequenced mRNA published in the same paper, five poly(A) tails attached to the 3′ genes were identified (Table 1 and S13). However, *TXNDC9-LYG1* did not seem to follow the proposed hypothesis. An inversion on Chr2:87–111 megabase pairs (Mbp) was identified by Breakdancer [34] (Table S14) and experimentally confirmed by Landry et al. [35]. Both parental genes are located within this region.

**Table 1:** Poly(A) tail occurrences of the parental genes in HeLa cells. For two fusion transcripts, *GFOD2-ENKD1* and *MFSD7-ATP5I*, no poly(A) tails were detected. Consistently, no tails were detected by Chang et al. [13] nor with an in-depth search in the TAIL-seq data

cis-SAGe/ gene fusion	Poly(A) tails	
(5′ gene - 3′ gene)	5′ gene	3′ gene
*FOXRED2-TXN2*	0	340
*LHX6-NDUFA8*	0	218
*SLC2A11-MIF*	0	842
*SLC45A3-ELK4*	0	5
*TXNDC9-LYG1*	103	0
*UBE2Q2-FBXO22*	0	97

The results shown in Table 1 confirm our assumption that a fusion transcript caused by a cis-SAGe mechanism or a chromosomal rearrangement lacks a poly(A) tail at the 5′ gene and instead has an elevated number of poly(A) tails attached to the 3′ gene.

#### Distinction among fusion transcripts caused by trans-splicing

In the case of a fusion transcript caused by trans-splicing, both parental genes were transcribed and poly-adenylated. Poly(A) tails for both parental genes were observed (Table 2).

**Table 2:** Poly(A) tails occurrences of the parental genes assumed to occur via trans-splicing in HeLa cells

Trans-splicing fusion transcript	Poly(A) tails	
(5′ gene - 3′ gene)	5′ gene	3′ gene
*DHRS13-FLOT2*	64	71
*TINF2-NEDD8*	35	69
*VMP1-RPS6KB1*	51	48

The transcription-induced chimera *VMP1-RPS6KB1* is assumed to occur via trans-splicing [33] in HeLa cells. Indeed, this event was confirmed with STfusion. The fusions *TINF2-NEDD8* and *DHRS13-FLOT2* were experimentally confirmed [31], but both parental genes were polyadenylated. This data suggests that these fusions are caused by a trans-splicing event. The latter fusion transcript, *DHRS13-FLOT2,* is suggested to be transcription induced [36], because no genetic cause could be identified.

### STfusion and C-scores applied to clinical tissue samples

Spatial transcriptomics data published by Berglund et al. [19] was used to localise the cis-SAGe *SLC45A3-ELK3*. In this study, 12 tissue sections taken from a patient with prostate cancer were analysed; each section harboured epithelial areas annotated as healthy, inflamed, prostatic intraepithelial neoplasia (PIN, a precancerous lesion), cancerous with a Gleason Score (Gs) 3 + 3, or cancerous with Gs 3 + 4. The Gs is a grading system used to classify the aggressiveness of prostate cancer, scales range from 1 (appears healthy) to 5 (appears abnormal). The total Gs is a combination of two grades, one each for the dominant and minor area [37]. The tissues harbour the cis-SAGe *SLC45A3-ELK4* (Tables S15-S17) which contributes to cell proliferation in prostate cancer [32], Two fusion variants were identified in the bulk RNA-sequenced tissue sections: *SLC45A3-ELK4* exon 4-exon 2 and *SLC45A3-ELK4* exon 5-exon 2 (Fig. S1, Table S18).

#### Fusion transcript localisation using STfusion and C-scores

The C-score measures the fold change in the numbers of poly(A) tails on the parental gene compared to the parental gene sample mean expression. A higher C-score indicates that the occurrence of the cis-SAGe *SLC45A3-ELK4* is likely. This difference was caused by the chimeric mRNA and elevated 3′ gene *ELK4* expression, which is defined by the promoter of the 5′ UTR of the 5′ gene *SLC45A3*. A low C-score, however, represents a large number of poly(A) tails for the 5′ gene *SLC45A3* compared to the sample mean *SLC45A3* expression. This data indicates the occurrence of the cis-SAGe is very unlikely. The C-score mirrored the likelihood of cis-SAGe absence or occurrence in a spot as well as the expression levels of *SLC45A3* or cis-SAGe *SLC45A3-ELK3,* respectively (Additional file 1).

**Fig. 2:**
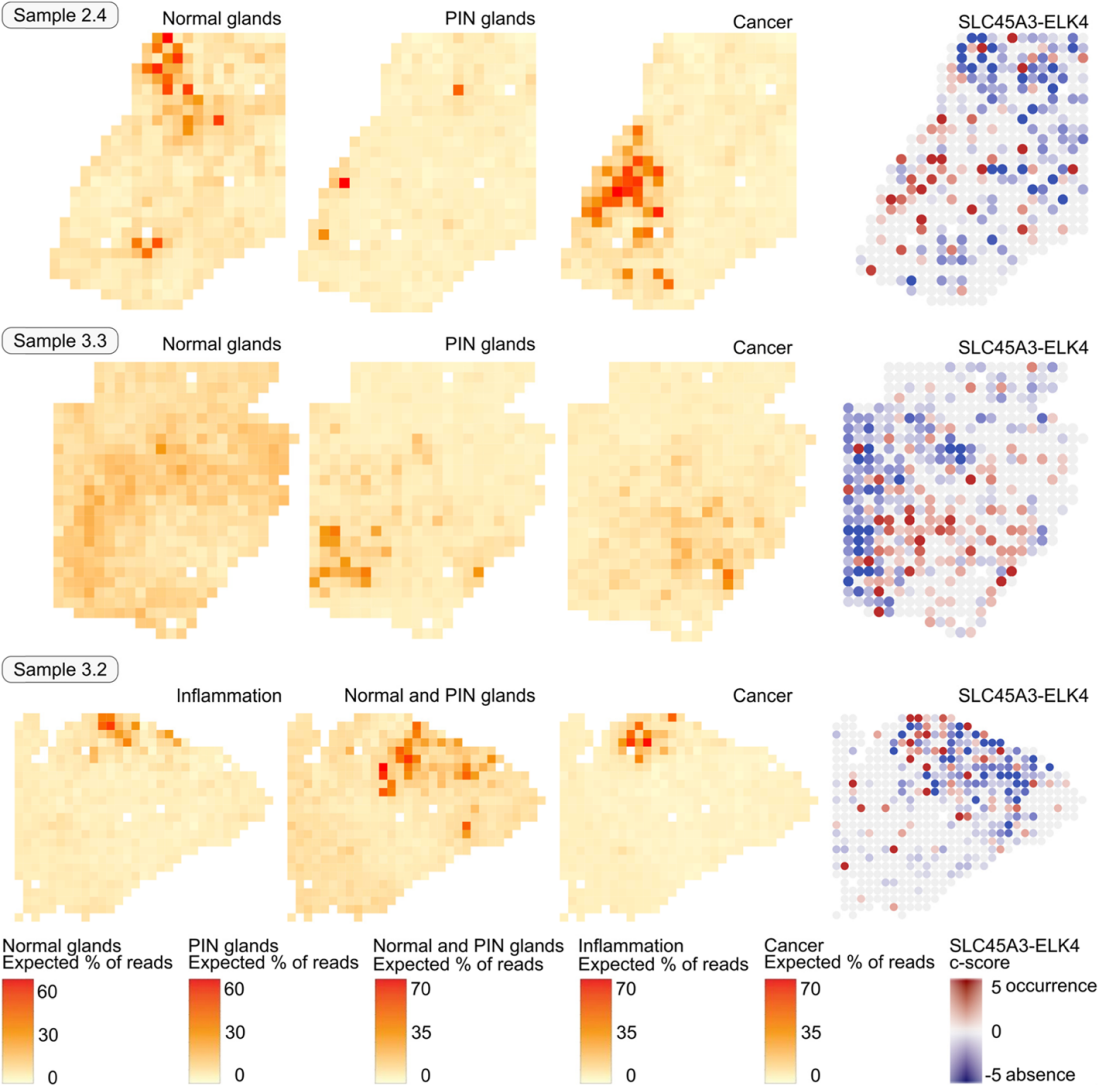
Activity maps of transcriptomic factors “Normal glands”, “PIN glands” and “Cancer” (from [19]) compared to the predicted occurrence of the fusion transcript SLC45A3-ELK4. The “Normal glands” factor is associated with absence of the fusion, while the “Cancer factor” is associated with occurrence of the fusion

The C-scores’ spatial distribution per sample was compared to the activity maps of the transcriptomic factors identified in the clinical tissue samples analysed with spatial transcriptomics (Figs. 2 and S2). The predicted occurrence of the cis-SAGe *SLC45A3-ELK4* in the 12 tissue sections was dominant in the centre or periphery of disease areas; the predicted absence of the cis-SAGe was dominant in normal glands.

The three spatial transcriptomics samples with clearly identifiable cancer areas and transcriptomic factors related to cancer, resulting from the joint STD analyses are shown in Fig. 2, together with the activity of selected factors and C-scores. For the transcriptomic factor “Cancer”, the predicted cis-SAGe occurs intensely at its activity centre in these samples, as well as at its periphery. In the cancer areas of samples 2.4 and 3.3 there are no spots with much higher expression of *SLC45A3* than *ELK4* (dark blue), hence no strong absence of the cis-SAGe is predicted in these areas. The cis -SAGe occurs only occasionally in the periphery of the PIN area in sample 3.3. Normal glands are dominated by absence of cis-SAGe. We note a few spots with strong cis-SAGe intensity scattered in various areas, often in the direct vicinity of spots with strong cis-SAGe absence.

To provide a statistical test for the coherence of disease areas and cis-SAGe occurrence, Spearman and Pearson correlations ρ were calculated (Tables 3 and S19, Fig. S3). The strongest correlation of cis-SAGe occurrence is to the cancerous areas in sample 2.4 (ρ _Pearson_ = 0.25, *p* = 1.07E-07), sample 3.3 (ρ _Pearson_ = 0.14, *p* = 1.55E-03), and sample 3.2 (ρ _Pearson_ = 0.12, *p* = 4.32E-03), and to the PIN areas in sample 2.4 (ρ _Spearman_ = 0.14, *p* = 3.15E-03), sample 1.2 (ρ _Pearson_ = 0.23, *p* = 4.22E-06), sample 4.1 (ρ _Pearson_ = 0.15, *p* = 1.17E-04), and sample 1.3 (ρ _Pearson_ = 0.13, *p* = 1.51E-03).

**Table 3:** Correlation of C-score and factor activities per sample shown in Fig. 2

Sample	Factor	Sample-wide				
		# spots	Correlation ρ _Spearman_	Correlation ρ _Pearson_	p-value for ρ _Spearman_	*p*-value for ρ _Pearson_
Sample 2.4	Normal glands	451	−0.10	− 0.13	4.19E-02	6.20E-03
Sample 2.4	PIN glands	451	0.14	0.02	3.15E-03	6.21E-01
Sample 2.4	Cancer	452	0.26	0.25	3.74E-08	1.07E-07
Sample 3.3	Normal glands	500	−0.02	−0.04	6.51E-01	3.53E-01
Sample 3.3	PIN glands	500	−0.03	0.00	4.91E-01	9.59E-01
Sample 3.3	Cancer	500	0.20	0.14	4.16E-06	1.55E-03
Sample 3.2	Inflammation	560	0.17	0.11	1.32E-04	1.50E-02
Sample 3.2	Normal & PIN glands	560	−0.17	−0.04	8.28E-05	3.86E-01
Sample 3.2	Cancer	560	0.16	0.12	2.61E-04	4.32E-03

**Fig. 3:**
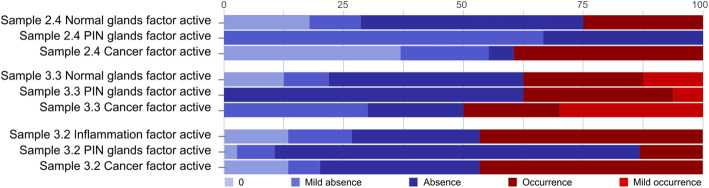
Fraction of spots with fusion transcript occurrence (C-score > 0) and absence (C-score ≤ 0) for the factors shown in Fig. 2. The factor activity threshold was set to 20%. Mild occurrence and mild absence C-score thresholds were set to 1 and − 1, respectively

The areas with active transcriptomic factors (“Normal glands”, “PIN glands”, “Inflammation”, and “Cancer”) were further analysed concerning the share of spots with predicted present or absent fusion transcripts (Fig. 3). In normal glands, the cis-SAGe is dominantly absent. In the cancerous areas of the sample 3.3, the share of spots with mild occurrence (0 < C-score < 1) is increased compared to the other factors, whereas in the PIN areas of the same samples, the share of spots with strong occurrence (C-score > 1) is increased.

#### Differentially expression and pathway annotation for cis-SAGe occurrence

The combination of spatial transcriptomics data and STfusion using C-scores offers new possibilities to explore fusion transcript occurrence, differences in cis-SAGe transcription levels and their spatial relation in clinical tissue samples.

**Fig. 4:**
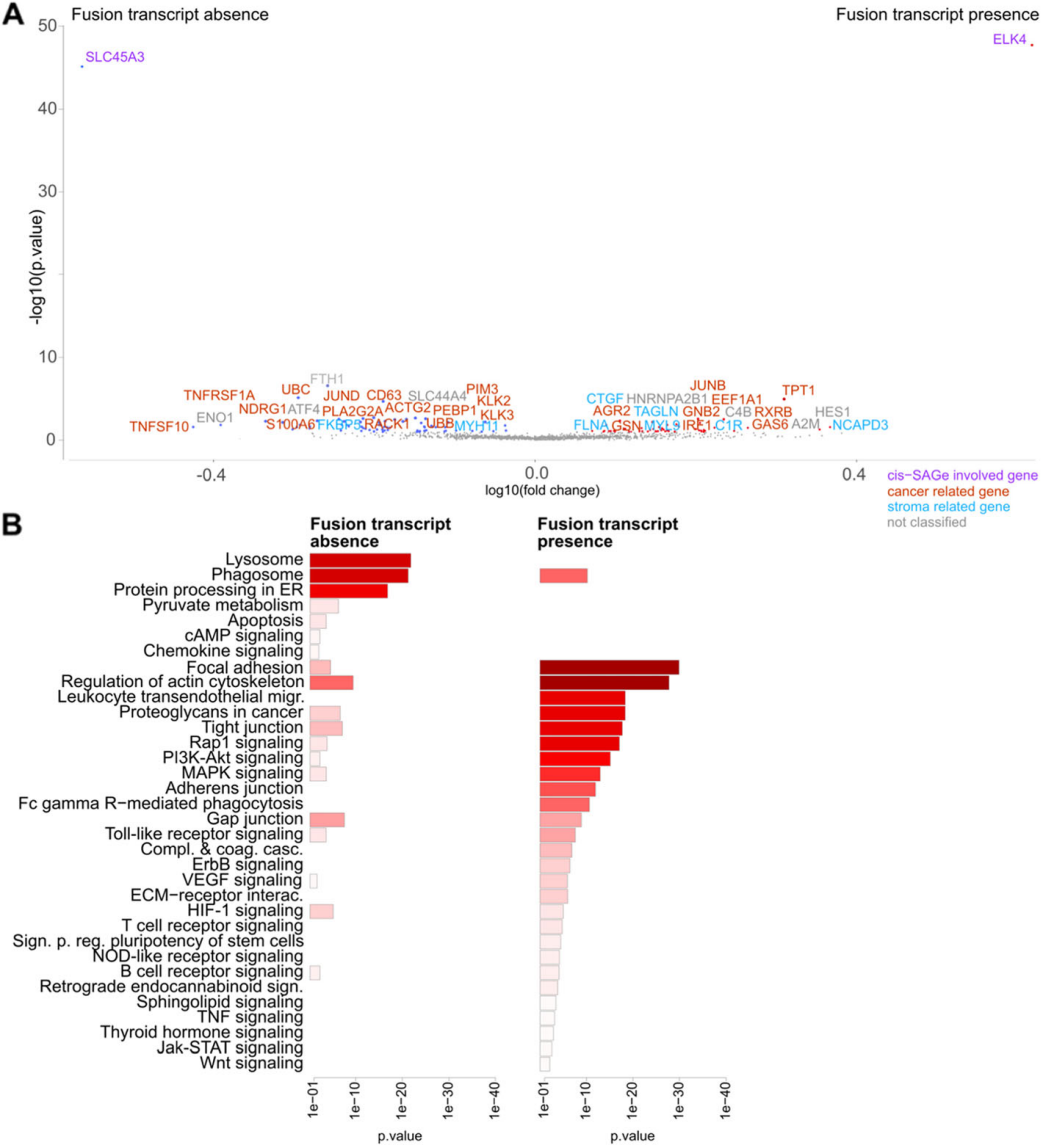
Differential expression and pathway annotation of cis-SAGe occurrence. Areas with absent and present fusion transcripts in sample 3.3 were compared. Besides normal glands, the sample harboured an area annotated as PIN and a large area annotated as cancerous of which some parts were annotated as aggressively cancerous (Gs 3 + 4). **a** Significantly differentially expressed genes (FDR, q < 0.1) are shown. **b** Significantly differentially expressed genes were submitted to PathwAX on the KEGG database. Enriched pathways are presented

Areas with absent and present *SLC45A3-ELK4* fusion transcripts were compared with regards to differentially and co-expressed genes and enriched pathways (Figs. 4, S5). In areas without fusion transcripts, there were pathways activated which are related to higher transcriptional stress (protein processing in the endoplasmic reticulum and lysosome). The pathways focal adhesion and regulation of actin cytoskeleton are highly active in the areas with cis-SAGe occurrence and are known to play a crucial role in cancer cell motility and invasion [38, 39]. Phosphatidylinositol-3-kinase (PI3K)-AKT signalling is linked to treatment resistance [40, 41].

#### Detectability of fusion transcript candidates

We found that the C-scores of fusion transcripts (cis-SAGe and gene fusions) identified in the 12 tissue sections are more dispersed than those calculated for random gene pairs. Based on a diversity index, true fusion transcripts are top ranked (Fig. S8).

To summarise the results, the proposed method, STfusion, identified fusion transcripts caused by a cis-SAGe mechanism or chromosomal rearrangement. It also distinguished these fusion transcripts from those caused by a trans-splicing event. Applying STfusion and the C-score to clinical tissue samples analysed with spatial transcriptomics demonstrated the spatial distribution of the fusion transcripts within the tissue section. Further, the fusion transcript was linked to the disease areas (inflammation, PIN, and cancer).

## Discussion

We propose a novel computational method to detect fusion transcripts. It is based on poly(A) tail presence or absence. We proved that a fusion transcript that lacks the poly(A) tail at the 5′ parental gene contains one at the 3′ gene. The number of poly(A) tails attached to the 3′ gene indicates the expression level of the fusion transcript defined by the 5′ gene promoter region. The novel method was verified on the chimeric transcripts caused by an incorrect cis-splicing of adjacent genes (cis-SAGe) mechanism or a chromosomal rearrangement (gene fusion) in HeLa cells. Fusion transcripts caused by trans-splicing with both genes that are poly-adenylated cannot be detected with the proposed method. However, our method helps to identify trans-splicing fusions among fusion transcripts identified with alternative methods (Tables 1 and 2).

The proposed method, STfusion, and the use of C-scores were applied to clinical tissue sections analysed with spatial transcriptomics. The tissue samples harbour areas annotated as inflammation, prostatic intraepithelial neoplastic and prostate cancer with different Gleason scores. Spatial transcriptomics, which uses the poly(A) tail as a proxy for expression levels, offers the opportunity to measure the unexpected amount of chimeric transcript parental gene expression levels at an almost single cell level. Fusion transcripts caused by cis-SAGe of the parental genes *SLC45A3* and *ELK4* were confirmed in the bulk sequenced RNA of the tissue sections. The identification of this fusion transcript in clinical tissues that harbour cancerous cells, and a spatial correlation to the disease areas was lacking. With the proposed method, we localised this fusion transcript in 12 tissue sections on almost single-cell level and detected a high variance of cis-SAGe expression in healthy and diseased areas which was reported earlier [42]. We showed the spatial expansion of the fusion transcript in the clinical samples and correlated the fusion transcript occurrence to areas annotated as diseased (Figs. 2 and S2, Table 3). Very high cis-SAGe expression levels were observed in the periphery or centre of the disease areas; in one sample, spots with very low fusion transcript expression levels were found in the cancerous area. Occasionally, the cis-SAGe occurred in normal gland areas. Very high *SLC45A3* expression increases the likelihood of the cis-SAGe *SLC45A3*-*ELK4* occurrence. This observation indicates that *SLC45A3*-*ELK4* occurrence is an early and local event in the course of prostate cancer development. It appears to commence with higher *SLC45A3* expression, continue with high cis-SAGe expression, and finally end in very low cis-SAGe expression.

Differentially expressed genes between areas with and without the fusion transcript were calculated and activated pathways inferred (Figs. 4 and S5). The observed activated pathways in areas of cis-SAGe occurrence correlated with disease progression. Further, we observed pathways related to higher transcriptional stress during the switch from high 5′ gene expression to high cis-SAGe expression.

The diversity of the C-scores measured with the diversity index can help to indicate fusion transcripts caused by a cis-SAGe mechanism or chromosomal rearrangement among random gene pairs.

The cause of a cis-SAGe occurrence has not yet been identified. Genetic rearrangement can be excluded, and thus epigenetic changes are the primary focus. There is a DNA motif sequence with (CCA)_n_ repetitions downstream of the intra-exonic fusion point of the 5′ gene *SLC45A3* that is linked to the i-Motif, a non-canonical DNA structure (Table S21). In more acidic environments, the motif sequence folds reversibly into an intramolecular intercalated cytosine tetraplex and thus can serve as a molecular switch [43–45]. This switch might be involved in passing over the stop codon of the terminal exon and thus the poly(A) tail signal for the 5′ gene.

## Conclusions

Fusion transcripts are detectable by their absent poly(A) tails for the 5′ gene and elevated number of poly(A) tails for the 3′ gene. The presented method, STfusion, uses this concept to detect fusion transcripts in data sets for which the number of poly(A) tails per gene is available. The method further distinguishes chimeric transcripts from chimeras caused by trans-splicing events and can localise fusion transcripts in clinical tissue samples at almost single cell level. It can also be used to identify novel fusion transcripts.

## Supplementary information

**Additional file 1.** ST_cis-SAGe ST read counts of the parental genes of the cis-SAGe SLC45A3-ELK4 and C-scores obtained in the clinical tissue samples analysed with Spatial Transcriptomics

**Additional file 2.** Fusion transcript detection using spatial transcriptomics [46–52].

## Data Availability

The code to run STfusion, to calculate C-scores and to plot the spatial distribution, is available on the Bitbucket repository https://bitbucket.org/sonnhammergroup/stfusion/. A Shiny application ‘STfusion’ was also built and is freely available at https://stfusion.shinyapps.io/stfusion_shiny/. The spatial transcriptomics datasets analysed in this study were obtained from doi:10.1038/S41467-018-04724-5, available on the spatial research repository https://www.spatialresearch.org/resources-published-datasets/10-1038-s41467-018-04724-5/. The sequenced reads using TAIL-seq of HeLa cells analysed during the current study are available in the NCBI Gene Expression Omnibus (GEO) database (accession number GSM1242325). These datasets were derived from the following public domain resources: https://www.ncbi.nlm.nih.gov/geo/query/acc.cgi?acc=GSM1242325. The human assembly GRCh38 Ensembl (release 84) files were downloaded from ftp://ftp.ensembl.org/pub/release-84/ .

## Abbreviations

cis-SAGe: cis-Splicing of Adjacent Genes
FDR: False discovery rate
FOSB: FBJ murine Osteosarcoma viral oncogene homolog B
Gs: Gleason score
Kbp: Kilo Base Pairs
MDS: Minimal detectable signal
nt: Nucleotide
PI3K: Phosphoinositide 3-kinase
R: Ratio
sd: Standard Deviation
STD: Spatial transcriptomics decomposition
