# Supplementary material for: Fusion transcript detection using spatial transcriptomics
Source: BMC Med Genomics. 2020 Aug 4;13:110. doi: 10.1186/s12920-020-00738-5 (PMC7437936; doi:10.1186/s12920-020-00738-5)
Supplement: Supplementary file 2 — Additional file 2. Fusion transcript detection using spatial transcriptomics [46–52]. [file 12920_2020_738_MOESM2_ESM.docx]

**Fusion transcript detection using spatial transcriptomics**

Supplementary

# The Spatial Transcriptomics method

Messenger RNA (mRNA) and the poly(A) tails are captured with an poly-T20VN oligonucleotide in each of the 1,007 features (spots) of the spatial transcriptomics array and tagged with a unique molecular identifier (UMI), and a spatial barcode. From the captured mRNA, complementary DNA is generated by reverse transcription and sequenced using paired-end sequencing. The first read of the pairs encloses barcode and UMI, the second read contains the genetic information. Finally, the resulting read counts for every gene in every spot is overlaid with annotated brightfield image of H&E-stained tissue sections for further data analysis. The number of captured poly(A) tails mirrors the gene expression level on almost single-cell level. The fragment of the sequence before the cleavage site (101 or 121 base pairs, bp) is mapped to a reference genome. For any correctly transcribed gene, the amount of poly(A) tail reflects the expression level. [18,19]

# The fusion transcripts in HeLa cancer cells

**Table S1. The cis-SAGe *LHX6-NDUFA8.*** Gene structure of parental genes *LHX6* and *NDUFA8.*

|  | ***LHX6*** | ***NDUFA8*** |
| --- | --- | --- |
| Strand | reverse | reverse |
| Gene | 5´ | 3´ |
| Chr | 9 | 9 |
| Start & stop position (GRCh38) | 122,185,652-122,229,626 | 122,132,466-122,159,819 |
| Length [bp] | 43,975 | 27,354 |

**Table S2. The cis-SAGe *SLC2A11-MIF.*** Gene structure of parental genes *SLC2A11* and *MIF.*

|  | ***SLC2A11*** | ***MIF*** |
| --- | --- | --- |
| Strand | forward | forward |
| Gene | 5´ | 3´ |
| Chr | 22 | 22 |
| Start & stop position (GRCh38) | 23,856,703-23,886,309 | 23,894,004-23,895,227 |
| Length [bp] | 29,607 | 1,224 |

**Table S3. The cis-SAGe *SLC45A3-ELK4.*** Gene structure of parental genes *SLC45A3* and *ELK4.*

|  | ***SLC45A3*** | ***ELK4*** |
| --- | --- | --- |
| Strand | reverse | reverse |
| Gene | 5´ | 3´ |
| Chr | 1 | 1 |
| Start & stop position (GRCh38) | 205,657,851-205,680,502 | 205,593,466-205,634,085 |
| Length | 22,608 | 24,019 |

**Table S4. The fusion transcript *FOXRED2-TXN2.*** Gene structure of parental genes *FOXRED2* and *TXN2.*

|  | ***FOXRED2*** | ***TXN2*** |
| --- | --- | --- |
| Strand | reverse | reverse |
| Gene | 5´ | 3´ |
| Chr | 22 | 22 |
| Start & stop position (GRCh38) | 36,487,186-36,507,101 | 36,467,036-36,482,030 |
| Length [bp] | 19,916 | 14,995 |

**Table S5. The fusion transcript *GFOD2-ENKD1.*** Gene structure of parental genes *GFOD2* and *ENKD1*.

|  | ***GFOD2*** | ***ENKD1*** |
| --- | --- | --- |
| Strand | revers | reverse |
| Gene | 5´ gene | 3´ |
| Chr | 16 | 16 |
| Start & stop position (GRCh38) | 67,674,531-67,719,421 | 67,662,945-67,667,265 |
| Length [bp] | 44,891 | 4,321 |

**Table S6. The fusion transcript *TXNDC9-LYG1.*** Gene structure of parental genes *LYG1* and *TXNDC9*.

|  | ***TXNDC9*** | ***LYG1*** |
| --- | --- | --- |
| Strand | reverse | reverse |
| Gene | 5´ | 3´ |
| Chr | 2 | 2 |
| Start & stop position (GRCh38) | 99,318,982-99,340,702 | 99,284,238-99,304,742 |
| Length [bp] | 21,721 | 20,505 |

**Table S7. The fusion transcript *MFSD7-ATP5I.*** Gene structure of parental genes *MFSD7* and *ATP5I*.

|  | ***MFSD7*** | ***ATP5I*** |
| --- | --- | --- |
| Strand | reverse | reverse |
| Gene | 5´ | 3´ |
| Chr | 4 | 4 |
| Start & stop position (GRCh38) | 674,690-689,468 | 672,436-674,338 |
| Length [bp] | 14,779 | 1,903 |

**Table S8. The fusion transcript *UBE2Q2-FBXO22.*** Gene structure of parental genes *UBE2Q2* and *FBXO22*.

|  | ***UBE2Q2*** | ***FBXO22*** |
| --- | --- | --- |
| Strand | forward | forward |
| Gene | 5´ | 5´ |
| Chr | 15 | 15 |
| Start & stop position (GRCh38) | 75,843,285-75,901,078 | 75,903,859-75,942,510 |
| Length [bp] | 57,794 | 38,652 |

**Table S9. The fusion transcript *DHRS13-FLOT2.*** Gene structure of parental genes *DHRS13* and *FLOT2.*

|  | ***DHRS13*** | ***FLOT2*** |
| --- | --- | --- |
| Strand | reverse | reverse |
| Gene | 5´ | 3´ |
| Chr | 17 | 17 |
| Start & stop position (GRCh38) | 28,897,781-28,903,071 | 28,879,335-28,897,697 |
| Length [bp] | 5,291 | 18,363 |

**Table S10. The fusion transcript *VMP1-RPS6KB1.*** Gene structure of parental genes *RPS6KB1* and *VMP1*.

|  | ***VMP1*** | ***RPS6KB1*** |
| --- | --- | --- |
| Strand | forward | forward |
| Gene | 5´ | 3´ |
| Chr | 17 | 17 |
| Start & stop position (GRCh38) | 59,707,192-59,842,255 | 59,893,046-59,950,564 |
| Length [bp] | 135,064 | 57,519 |

**Table S11. The fusion transcript *TINF2-NEDD8.*** Gene structure of parental genes *TINF2* and *NEDD8*.

|  | ***TINF2*** | ***NEDD8*** |
| --- | --- | --- |
| Strand | reverse | reverse |
| Gene | 5´ | 3´ |
| Chr | 14 | 14 |
| Start & stop position (GRCh38) | 24,234,403-24,243,027 | 24,216,851-24,232,454 |
| Length [bp] | 8,625 | 15,604 |

# Method details of STfusion

**Table S12.** Examples of Poly(A) tail occurrence or absence. For the C-score calculation, the normal (mean) number of poly(A) tails for the 5’ gene was set to 100 and for the 3’ gene was set to 10.

| Case | # Poly(A) tails 5’ gene | # Poly(A) tails 3’ gene | C-score |
| --- | --- | --- | --- |
| 1a. Fusion transcript absence   - normal transcription of both parental genes | 100 | 10 | -1.0 |
| 1b. Fusion transcript absence   - low transcription of both parental genes | 50 | 5 | -0.5 |
| 1c. Fusion transcript absence   - Higher transcription of both parental genes | 400 | 40 | -4.0 |
| 1d. Fusion transcript absence   - Higher transcription of the 5’ parental gene solely | 400 | 10 | -4.0 |
| 2a. Fusion transcript presence   - transcription of the fusion transcript; transcription level is defined by the promoter region of the 5’ gene (as in 1a) | 0 | 100 | 10.0 |
| 2b. Fusion transcript presence   - Low transcription of the fusion transcript; transcription level is defined by the promoter region of the 5’ gene (as in 1b) | 0 | 50 | 5.0 |
| 2c. Fusion transcript presence   - High transcription of the fusion transcript; transcription level is defined by the promoter region of the 5’ gene (as in 1c and 1d) | 0 | 400 | 40.0 |

## Method verification in HeLa cancer cells

**Table S13. Poly(A) tail occurrence in HeLa using paired-end sequenced mRNA produced by TAIL-seq [13].** This table contains the poly(A) tails found with an in-depth search shown in Table 1.

| Cis-SAGe | Gene | Poly(A) tail read | Read name | Chr | Start GRCh38  [bp] | MAPQ  (aligner) |
| --- | --- | --- | --- | --- | --- | --- |
| *SLC45A3-ELK4* | *SLC45A3* | none |  |  |  |  |
|  | *ELK4* | Yes | SRR1005384.2369110 | 1 | 205,607,958 | 50 (Tophat2 [15]) |
|  | *ELK4* | yes | SRR1005384.7994172 | 1 | 205,608,026 | 255 (STAR [16]) |
|  | *ELK4* | yes | SRR1005384.5120472 | 1 | 205,615,230 | 255 (STAR) |
|  | *ELK4* | yes | SRR1005384.709104 | 1 | 205,619,279 | 50 (Tophat2) |
|  | *ELK4* | yes | SRR1005384.7257115 | 1 | 205,619,300 | 50 (Tophat2) |
| *GFOD2-ENKD1* | *GFOD2* | none |  |  |  |  |
|  | *ENKD1* | none |  |  |  |  |
| *MFSD7-ATP5I* | *MFSD7* | none |  |  |  |  |
|  | *ATP5I* | none |  |  |  |  |

## Inversions identified in HeLa cells

**Table S14. Inversions in Hela cancer cells identified using Breakdancer [34] against GRCh38.**

| Chr1 | Pos1  [Mbp ] | Chr2 | Pos2  [Mbp] | Inversion size | Score | Number of reads |
| --- | --- | --- | --- | --- | --- | --- |
| 1 | 80.3 | 1 | 80.3 | 703 | 90 | 2 |
| 1 | 207.1 | 1 | 207.1 | 251 | 87 | 2 |
| 1 | 230.5 | 1 | 230.5 | 1,614 | 99 | 2 |
| 2 | 87.2 | 2 | 111.7 | 24,491,189 | 85 | 2 |
| 2 | 87.5 | 2 | 111.5 | 23,961,213 | 94.5 | 4 |
| 2 | 87.6 | 2 | 111.3 | 23,690,047 | 85 | 2 |
| 2 | 110.0 | 2 | 110.4 | 393,771 | 99 | 2 |
| 2 | 179.3 | 2 | 195.7 | 16,391,275 | 92 | 2 |
| 3 | 43.8 | 3 | 43.8 | 711 | 99 | 4 |
| 3 | 44.7 | 3 | 44.7 | 1,258 | 83 | 2 |
| 4 | 87.9 | 4 | 87.9 | 11,153 | 96 | 2 |
| 5 | 0.2 | 5 | 1.6 | 1,386,092 | 99 | 2 |
| 5 | 11.6 | 5 | 11.6 | 764 | 87 | 2 |
| 5 | 20.7 | 5 | 34.1 | 13,377,479 | 90 | 2 |
| 5 | 20.9 | 5 | 34.3 | 13,398,338 | 88 | 6 |
| 5 | 28.9 | 5 | 28.9 | 1,664 | 99 | 8 |
| 5 | 148.2 | 5 | 148.2 | 668 | 99 | 10 |
| 6 | 89.2 | 6 | 89.2 | 586 | 99 | 5 |
| 6 | 130.5 | 6 | 130.5 | 3,537 | 99 | 3 |
| 7 | 67.2 | 7 | 72.6 | 5,469,533 | 98 | 2 |
| 7 | 143.6 | 7 | 143.8 | 233,734 | 90 | 2 |
| 7 | 150.2 | 7 | 153.8 | 3,656,988 | 98 | 2 |
| 8 | 7.4 | 8 | 7.9 | 532,698 | 94 | 2 |
| 10 | 4.9 | 10 | 5.0 | 81,426 | 85 | 2 |
| 11 | 2.9 | 11 | 102.0 | 99,130,281 | 86 | 2 |
| 11 | 28.9 | 11 | 61.3 | 32,333,538 | 85 | 2 |
| 11 | 37.8 | 11 | 81.2 | 43,355,730 | 99 | 3 |
| 11 | 80.9 | 11 | 85.8 | 4,823,529 | 99 | 4 |
| 12 | 71.1 | 12 | 71.1 | 397 | 99 | 2 |
| 12 | 71.3 | 12 | 71.3 | 526 | 99 | 2 |
| 14 | 85.2 | 14 | 85.2 | 2,080 | 82 | 2 |
| 15 | 30.4 | 15 | 32.2 | 1,787,699 | 85 | 2 |
| 15 | 30.8 | 15 | 31.7 | 903,270 | 92 | 2 |
| 17 | 15.5 | 17 | 18.8 | 3,294,643 | 99 | 2 |
| 18 | 13.0 | 18 | 13.0 | 1,919 | 88 | 2 |
| 21 | 26.6 | 21 | 26.6 | 1,002 | 94 | 4 |

# cis-SAGe *SLC45A3-ELK4* in the clinical tissue samples

The cis-SAGe *SLC45A3-ELK*4 was first identified by Rickman et al. [46]. He suspected it to be caused “through a mechanism other than chromosomal rearrangement”. Zhang et al. [32] proved that *SLC45A3-ELK4* is a cis-splicing fusion transcript with a premature RNA transcribed across the gene boundarie. Exons belonging to the parental genes *SLC45A3* and *ELK4* are spliced together. Its level of expression correlates with disease progression. Rickman et al. [46] found the highest levels in prostate cancer metastases. The fusion transcript of *SLC45A3-ELK4* is a mixture of two pre-mRNA sequences, its level of transcription is defined by the transcription factors of the 5´ gene *SLC45A3* which is higher expressed in prostate tissues. However, only the *ELK4* fragment is translated into a protein [7]. This fact becomes increasingly interesting if both genes should have been transcribed at different expression levels and one has an importing role in regulating cell proliferation as it is the case for *ELK4*. Moreover, *ELK4* is Androgen Receptor (*AR*) regulated and makes it thus to a potential target for cancer treatment [46,47].

**Table S15. The cis-SAGe *SLC45A3-ELK*4.** Gene structures of parental genes *SLC45A3* and *ELK4* (GRCh38)

|  | ***SLC45A3*** | ***ELK4*** |
| --- | --- | --- |
| TSS | 205,680,459 (1.rf) | 205,631,962 (2.rf) |
| Promoter | 205,681,001-205,679,601 | 205,632,601-205,630,200 |
| Cis-regulatory elements   - TATA box - Kozak signal - TF binding site I - TF binding site II - *CTCF* binding site I - *CTCF* binding site II - Enhancer I - Enhancer II - Enhancer III | none  205,658,602-205,659,402  205,657,402-205,658,202 | none |
| Exons   - Exon 1 - Exon 2 - Exon 3 - Exon 4 - Exon 5 | 205,680,459-205,680,394  205,664,886-205,664,485  205,663,618-205,662,833  205,662,126-205,661,861  205,659,671-205,657,851 | 205,631,962-205,631,632  205,623,891-205,623,676  205,620,838-205,619,966  205,619,073-205,618,957  205,616,644-205,607,943 |
| Start codon in initial exon (AUG) | 205,664,648 (2.rf) | 205,623,882 (1.rf) |
| Stop codon in terminal exon (TAG,TAA,TGA) | 205,659,228 (3.rf) | 205,616,549 (2.rf) |
| Poly (A) signals  (AATAAA) | 205,658,856 (1.rf) | 205,607,965 (2.rf)  205,608.999 (1.rf)  205,613,620 (2.rf) |
| Cleavage site | 205,657,851 (3.rf) | 205,607,943 (3.rf) |

## *SLC45A3-ELK4* confirmation in bulk sequenced RNA of the clinical samples

**Table S16. Encompassing and spanning reads in the alignment using Tophat2**. In total, the cis-SAGe could be confirmed in 8 of the 12 tissue samples. The samples 1.1, 2.3, 3.1, and 4.1, where the cis-SAGe could not be identified in bulk sequenced data, a very low coverage at the two fusion points was observed.

| Sample | # reads at 1:205,659,488 | # reads at 1:205,661,860 | # reads at 1:205,623,890 | # encom- passing reads | # spanning reads | #variants detected with Fusioncatcher [28] |
| --- | --- | --- | --- | --- | --- | --- |
|  | *SLC45A3* | *SLC45A3* | *ELK4* | *SLC45A3 -ELK4* | *SLC45A3 -ELK4* |  |
| 1.1 | 88 | 149 | 4 | 0 | 0 |  |
| **1.2** | 1149 | 2353 | 27 | 2 | 12 | 2 |
| **1.3** | 155 | 244 | 14 | 2 | 4 | 2 |
| 2.1 | 125 | 173 | 7 | 1 | 2 | 2 |
| 2.3 | 70 | 93 | 2 | 0 | 0 |  |
| **2.4** | 832 | 1528 | 15 | 1 | 2 | 1 |
| 3.1 | 76 | 15 | 12 | 0 | 0 |  |
| 3.2 | 165 | 272 | 8 | 1 | 4 | 1 |
| **3.3** | 1538 | 2753 | 17 | 3 | 9 | 2 |
| 4.1 | 57 | 79 | 1 | 0 | 0 |  |
| 4.2 | 123 | 172 | 7 | 1 | 2 | 1 |
| 4.3 | 168 | 291 | 15 | 2 | 8 | 2 |

**Table S17. Encompassing and spanning reads in the alignment using STAR.** The cis-SAGe could be confirmed in 9 of the 12 tissue samples, one more sample as in the alignment using Tophat2 (Table S18). This additional sample 2.3 had one encompassing read.

| Sample | # reads at 1:205,659,488 | # reads at 1:205,661,860 | # reads at 1:205,623,890 | # encom- passing reads | # spanning reads | # variants detected with Fusioncatcher |
| --- | --- | --- | --- | --- | --- | --- |
|  | *SLC45A3* | *SLC45A3* | *ELK4* | *SLC45A3 -ELK4* | *SLC45A3 -ELK4* |  |
| 1.1 | 184 | 329 | 5 | 0 | 0 |  |
| **1.2** | 875 | 1598 | 21 | 9 | 8 | 2 |
| **1.3** | 803 | 1227 | 18 | 5 | 4 | 2 |
| 2.1 | 334 | 508 | 10 | 4 | 4 | 2 |
| 2.3 | 110 | 177 | 2 | 1 | 0 |  |
| **2.4** | 612 | 1035 | 13 | 1 | 3 | 1 |
| 3.1 | 153 | 260 | 14 | 0 | 0 |  |
| 3.2 | 1063 | 1699 | 11 | 5 | 6 | 1 |
| **3.3** | 871 | 1474 | 17 | 8 | 7 | 2 |
| 4.1 | 85 | 155 | 3 | 0 | 0 |  |
| 4.2 | 341 | 577 | 8 | 2 | 2 | 1 |
| 4.3 | 1365 | 2110 | 19 | 10 | 7 | 2 |

## *SLC45A3-ELK4* variants

**Figure S1.** **Cis-SAGe *SLC45A3-ELK4* variants.** Two fusion variants were detected in bulk sequenced RNA of the prostate tissue samples: two fusion points in *SLC45A3* on Chr1:205661860 (exon 4) and Chr1:205659488 (exon 5) and one fusion point in *ELK4* on chr1:205623890 (exon 2).


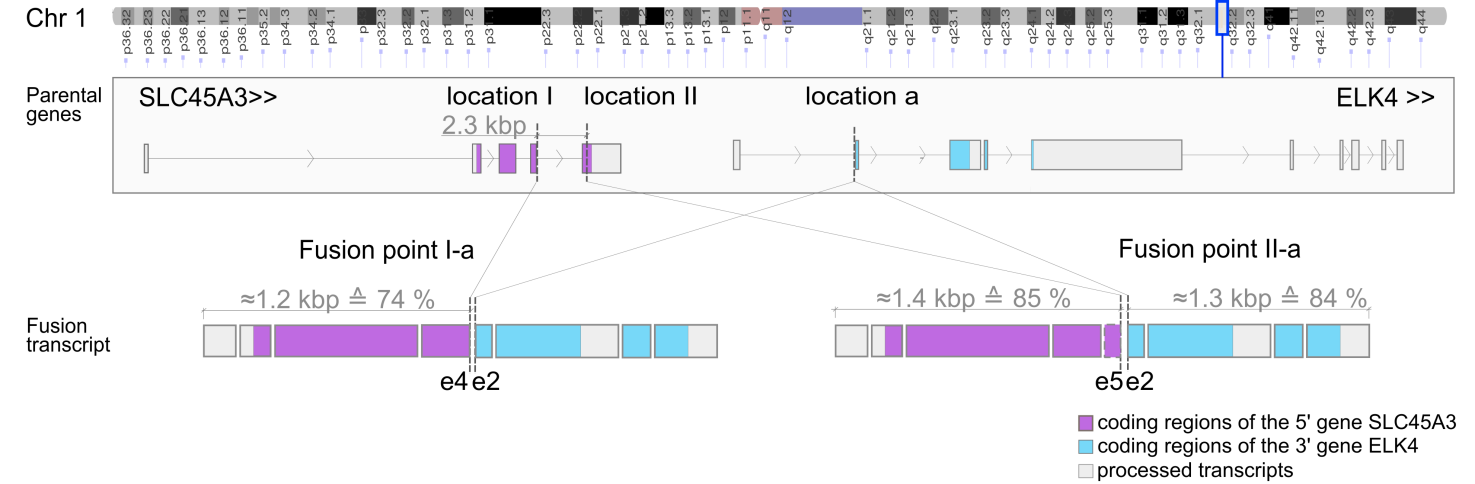


**Table S18. Cis-SAGe *SLC45A3-ELK4* variants in the 12 clinical tissue samples.**

| Fusion points | *SLC45A3* | *ELK4* | Distance |
| --- | --- | --- | --- |
| - I-a e4e2      - II-a e5e2 | - I Chr1:205,661,860   (after exon 4)   - II Chr1:205,659,488   (within exon 5) | - a Chr1:205,623,890   (before exon 2) | 37,970 bp  35,598 bp |

## *SLC45A3-ELK4* localisation in the remaining clinical tissue samples


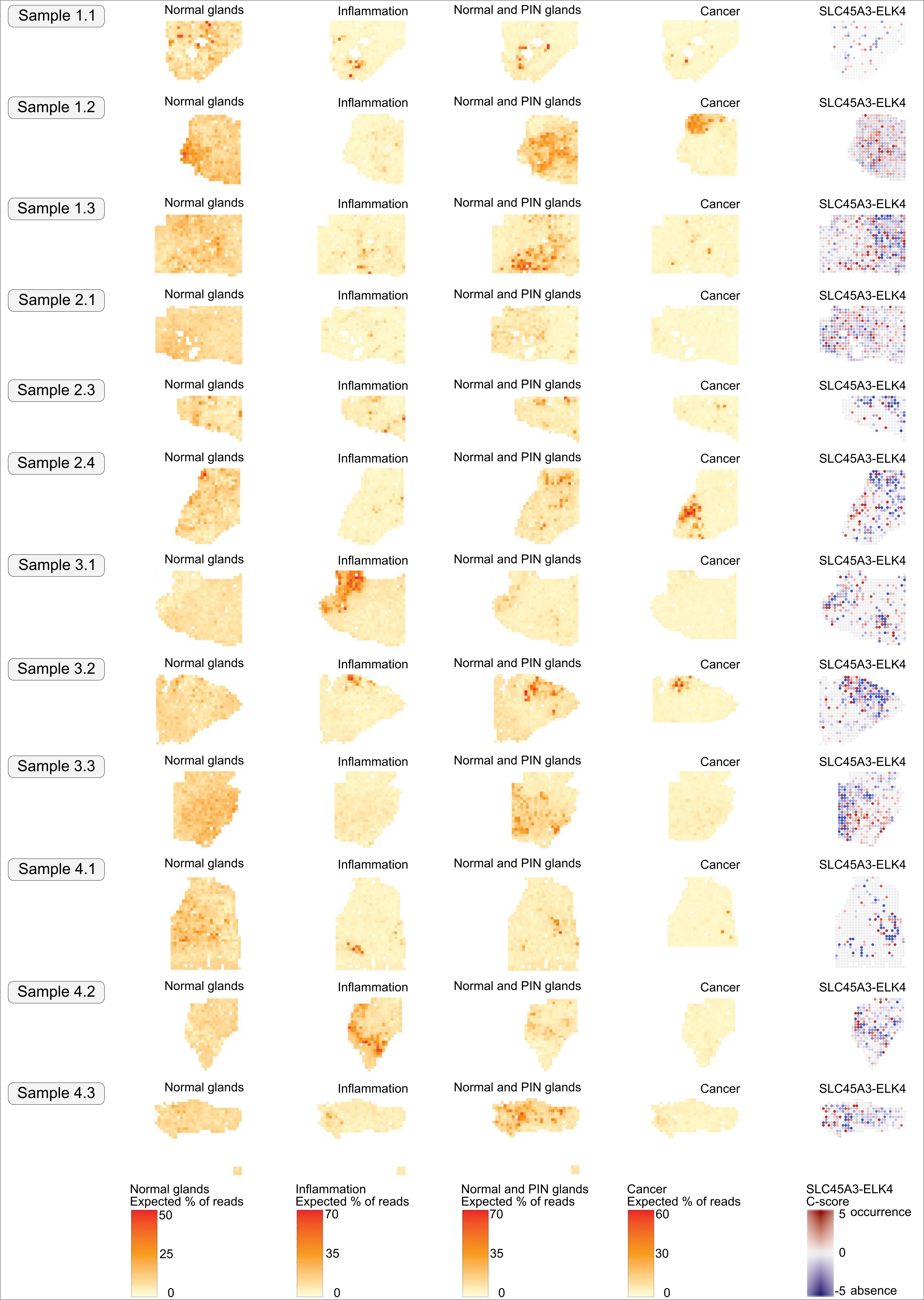


**Figure S2.** Activity maps for transcriptomics factors “Normal glands”, “Inflammation”, “Normal and PIN glands” and “Cancer” (from [19]) were compared to cis-SAGe *SLC45A3-ELK4* occurrence. The factor activity maps were based on a joined approach for 12 samples.

## Correlations of C-scores and factor activities


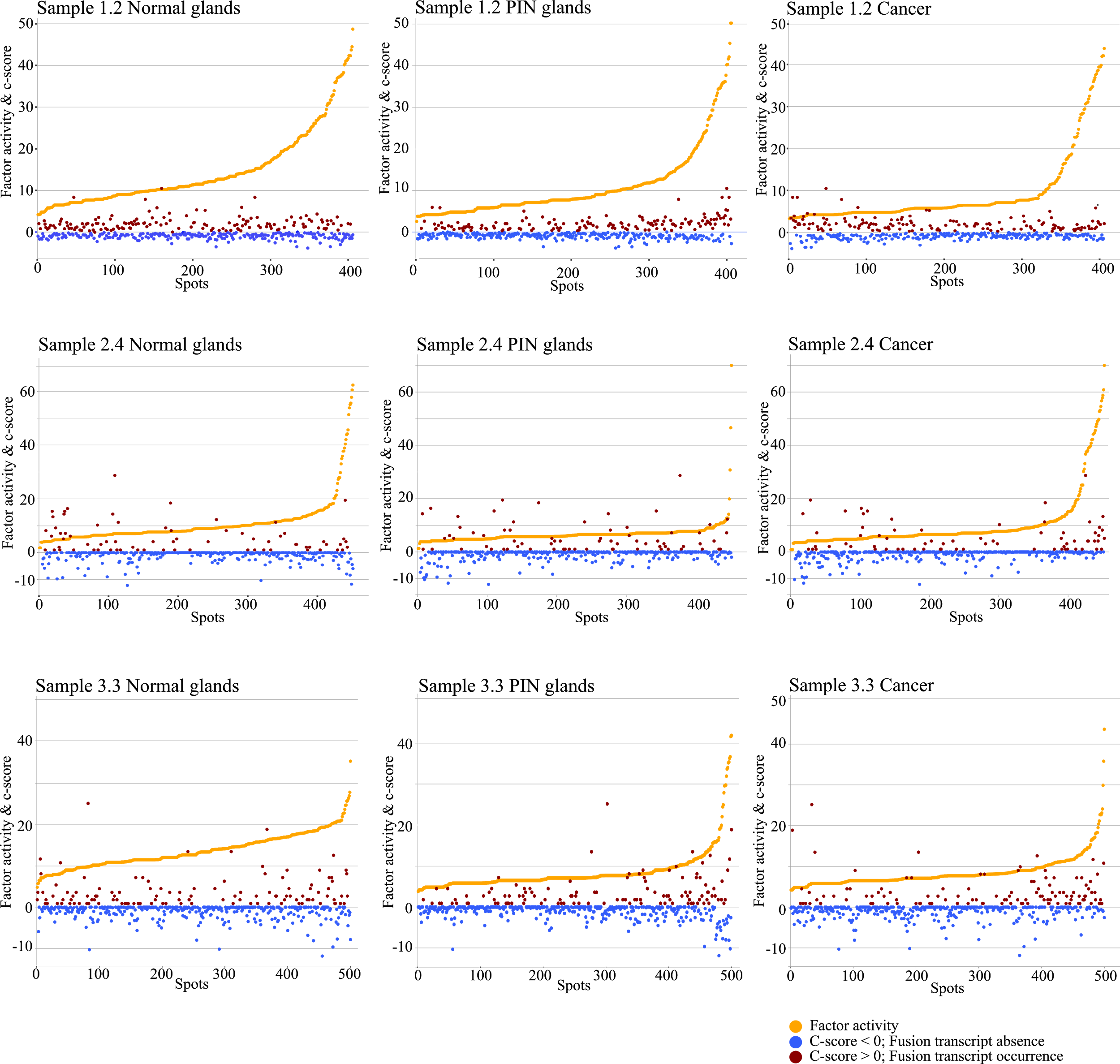


**Figure S3.** For the cancer samples 2.4, and 3.3, C-scores and factor activities (y-axis) per spot (x-axis) are shown.

**Table S19.** Spearman and Pearson correlation of C-score and factor activities. The factor activities were based on a joined approach for 12 samples as shown in Figure S2.

| Sample | Factor | Sample-wide | | | | |
| --- | --- | --- | --- | --- | --- | --- |
|  |  | # spots | Correlation  ⍴ _Spearman_ | Correlation  ⍴ _Pearson_ | p-value for  ⍴ _Spearman_ | p-value for  ⍴ _Pearson_ |
| Sample 1.1 | Cancer | 459 | 0.06 | 0.04 | 2.03E-01 | 4.07E-01 |
| Sample 1.1 | Inflammation | 460 | 0.02 | 0.03 | 6.05E-01 | 5.07E-01 |
| Sample 1.1 | PIN | 457 | -0.04 | -0.18 | 3.84E-01 | 1.74E-04 |
| Sample 1.2 | Cancer | 406 | -0.01 | -0.01 | 8.38E-01 | 8.50E-01 |
| Sample 1.2 | Inflammation | 406 | 0.09 | 0.07 | 7.38E-02 | 1.88E-01 |
| Sample 1.2 | PIN | 406 | 0.02 | 0.23 | 6.29E-01 | 4.22E-06 |
| Sample 1.3 | Cancer | 655 | 0.03 | 0.13 | 3.97E-01 | 8.92E-04 |
| Sample 1.3 | Inflammation | 655 | 0.08 | 0.04 | 5.41E-02 | 3.39E-01 |
| Sample 1.3 | PIN | 655 | 0.01 | 0.13 | 8.10E-01 | 1.51E-03 |
| Sample 2.1 | Cancer | 558 | -0.07 | 0.04 | 1.28E-01 | 3.36E-01 |
| Sample 2.1 | Inflammation | 559 | -0.01 | 0.07 | 8.55E-01 | 9.84E-02 |
| Sample 2.1 | PIN | 559 | -0.15 | 0.02 | 3.13E-04 | 6.15E-01 |
| Sample 2.3 | Cancer | 229 | 0.15 | 0.05 | 2.38E-02 | 4.94E-01 |
| Sample 2.3 | Inflammation | 229 | 0.16 | 0.02 | 1.70E-02 | 8.18E-01 |
| Sample 2.3 | PIN | 229 | -0.12 | -0.32 | 8.02E-02 | 1.43E-06 |
| Sample 2.4 | Cancer | 464 | 0.24 | 0.24 | 1.70E-07 | 2.29E-07 |
| Sample 2.4 | Inflammation | 464 | 0.08 | -0.01 | 9.64E-02 | 7.89E-01 |
| Sample 2.4 | PIN | 464 | -0.10 | -0.04 | 3.39E-02 | 3.81E-01 |
| Sample 3.1 | Cancer | 649 | 0.10 | 0.14 | 8.68E-03 | 6.78E-04 |
| Sample 3.1 | Inflammation | 651 | -0.04 | 0.06 | 3.40E-01 | 1.55E-01 |
| Sample 3.1 | PIN | 649 | -0.04 | -0.20 | 2.79E-01 | 4.61E-07 |
| Sample 3.2 | Cancer | 560 | 0.16 | 0.12 | 2.61E-04 | 4.32E-03 |
| Sample 3.2 | Inflammation | 560 | 0.17 | 0.11 | 1.32E-04 | 1.50E-02 |
| Sample 3.2 | PIN | 560 | -0.17 | -0.04 | 8.28E-05 | 3.86E-01 |
| Sample 3.3 | Cancer | 525 | 0.18 | 0.14 | 4.10E-05 | 1.84E-03 |
| Sample 3.3 | Inflammation | 525 | 0.02 | 0.08 | 6.96E-01 | 7.26E-02 |
| Sample 3.3 | PIN | 525 | -0.05 | 0.02 | 2.91E-01 | 6.84E-01 |
| Sample 4.1 | Cancer | 731 | -0.05 | 0.10 | 2.33E-01 | 8.56E-03 |
| Sample 4.1 | Inflammation | 731 | -0.09 | -0.02 | 1.44E-02 | 5.13E-01 |
| Sample 4.1 | PIN | 731 | 0.00 | 0.15 | 9.88E-01 | 1.17E-04 |
| Sample 4.2 | Cancer | 358 | 0.05 | 0.09 | 4.14E-01 | 1.14E-01 |
| Sample 4.2 | Inflammation | 358 | 0.02 | 0.06 | 6.90E-01 | 2.77E-01 |
| Sample 4.2 | PIN | 358 | 0.03 | 0.06 | 6.36E-01 | 2.55E-01 |
| Sample 4.3 | Cancer | 342 | 0.24 | 0.18 | 2.46E-05 | 2.23E-03 |
| Sample 4.3 | Inflammation | 342 | 0.14 | 0.07 | 1.56E-02 | 2.00E-01 |
| Sample 4.3 | PIN | 342 | -0.06 | 0.10 | 2.89E-01 | 7.07E-02 |

## C-score distributions for the 12 clinical tissue samples


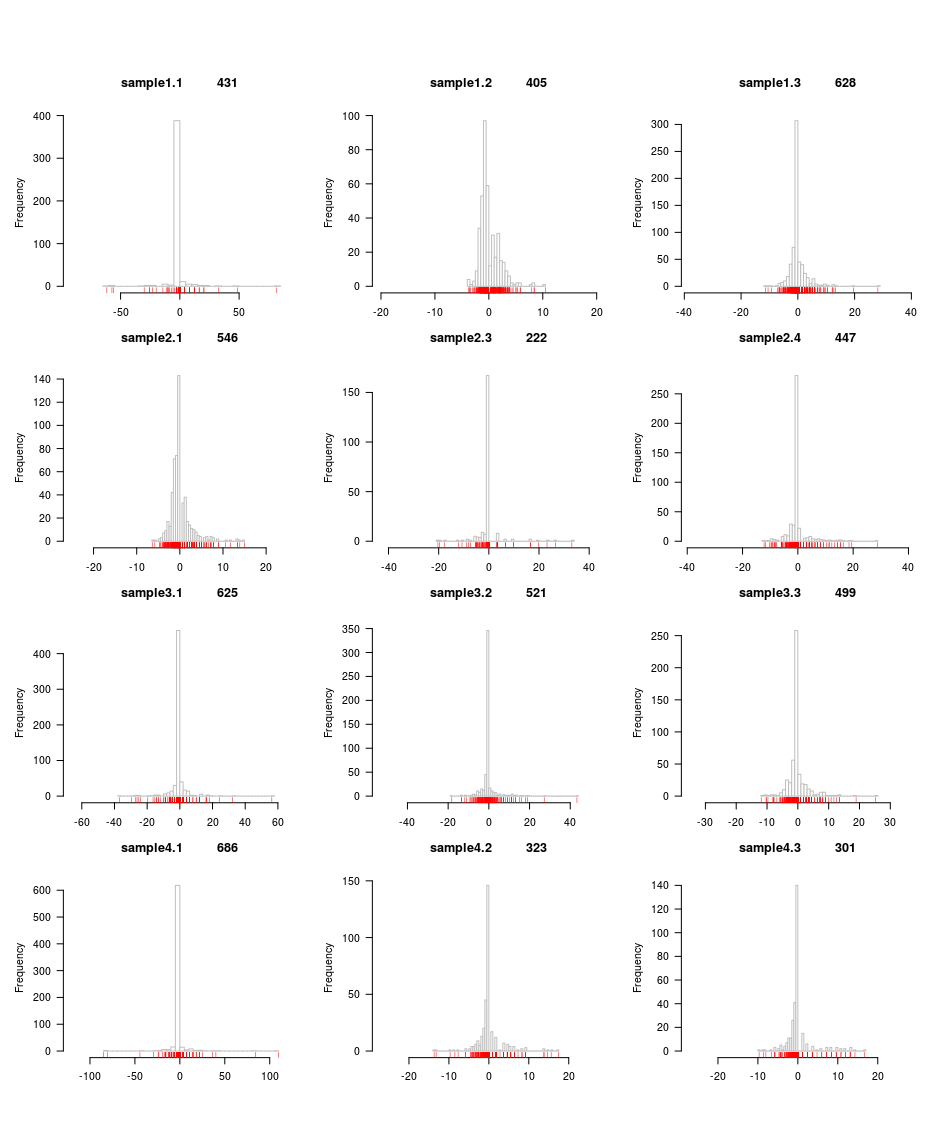


**Figure S4.** Distribution of C-scores per sample (comparison to Figure S7 showing C-scores with pseudocount of 1). Gray bars indicate the number of C-score observations per C-score value, red lines at the y-axis indicate the data points.

## Differentially expressed genes and pathway annotation

For an in-depth comparison of different intensities of a fusion transcript *SLC45A3-ELK4* occurrence or absence in sample 1.2, the sample with the highest data quality among the tissue samples, three comparisons were performed (Figure S3):

(i) Mild absence versus strong absence

(ii) Strong absence versus strong occurrence

(iii) Strong occurrence versus mild occurrence

To assign a spot to the groups “strong” or “mild”, these C-score thresholds were applied regardless of an annotation as stroma or epithelial:

(i) Strong absence ˗2.0 < C˗score

(ii) Mild absence ˗1.0 < C˗score < 0

(iii) Mild occurrence   0 < C˗score <1.0

(iv) Strong occurrence  2.0 < C˗score

**Figure S5.** Differential expression and pathway annotation of cis-SAGe occurence for sample 1.2. Epithelial areas are dominant in this sample [19]. **A** Depending on the C-score, four groups of spots were defined. Each of the four groups represents two levels (mild, strong) of cis-SAGe occurrence or absence. **B - D** Significantly differentially expressed genes (FDR, q < 0.1) obtained from the comparisons of the four groups are presented. Significantly differentially expressed genes were submitted to PathwAX on the KEGG database. Enriched pathways are shown. **B** DE analysis and pathway annotation for the comparison of mild absence and strong absence. **C** DE analysis and pathway annotation for the comparison of strong absence and strong occurrence. **D** DE analysis and pathway annotation for the comparison of strong occurrence and mild occurrence.


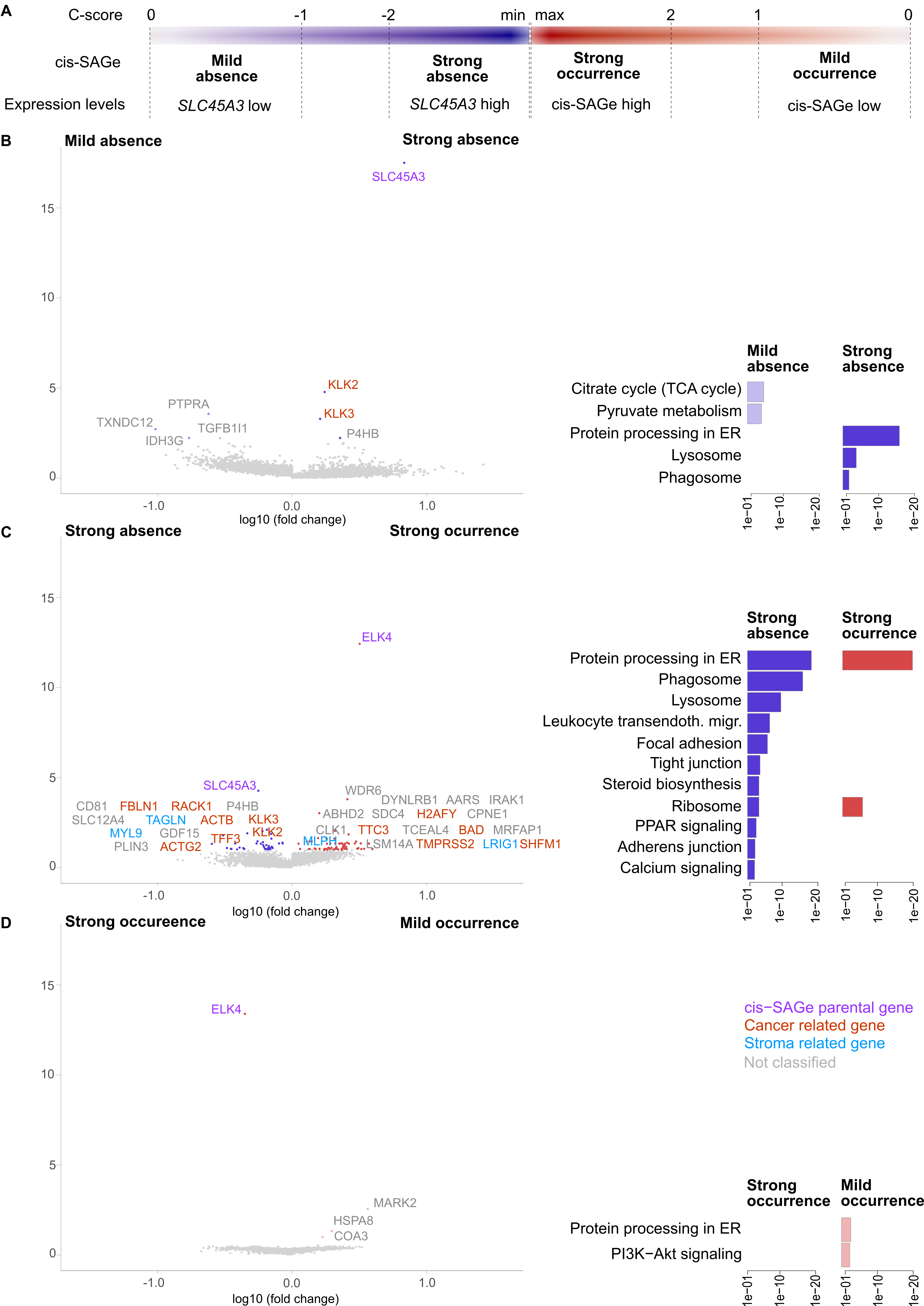


**Comparison of mild absence and strong absence**

In spots with low *SLC45A3* expression (light blue), the genes *PTPRA* and *TGFB1I1* are upregulated. *PTPRA* is a signalling molecule involved in cell growth, proliferation and oncogenic transformation [48]. *TGFB1I1* is a coactivator of AR and thus, plays a role in prostate cancer progression [49]. Notably, in the group of strong absence (dark blue), *KLK3* and *KLK2* are co-expressed with *SLC45A3*. Both genes, *KLK3* and *KLK2*, are *AR*-regulated and often upregulated in prostate cancer [50]. The activated pathway Citrate cycle (TCA cycle) is a typical phenomena of malignant transformation in prostate cancer mirroring a shift towards citrate oxidation to meet the higher energy demands of prostate cancer cells [51]. Areas of strong cis-SAGe absence (dark blue) show higher activation of the pathways protein processing in endoplasmic reticulum and lysosome. The pathway lysosome comprises digestive processes of macromolecules [52]. This indicates higher transcriptional stress, elevated levels of misfolded proteins and misfolded protein digestion.

**Comparison of strong absence and strong occurrence**

Significantly upregulated genes in areas with strong absence are *RACK1*, *ACTG2*, *KLK3* and *KLK2*. These genes are also significantly upregulated in areas with absent *SLC45A3-ELK4* in sample 3.3 (Figure 4). The pathway protein processing in endoplasmic reticulum is activated in both areas with strong absence and strong occurrence. As in Figure 4, the pathways Lysosome and Phagosome are activated in areas of strong absence. The pathway phagosome is linked to inflammation [53].

**Comparison of strong occurrence and mild occurrence**

In areas with mild cis-SAGe occurrence, only three significantly upregulated genes were observed, i.e. *MARK2*, *HSPA8* and *COA3*. The pathways protein processing in endoplasmic reticulum and PI3K-Akt signalling are slightly activated. The latter is linked to disease progression, poor outcome and resistance to androgen deprivation therapy [41].

## C-score with pseudo count

To avoid divisions of 0, a pseudo count of 1 can be added to both dividend and divisor of the ratios R_5´_ and R_3´_. The distribution of the C-scores then changes slightly which can be circumvented using the minimal detectable signal (MDS) as threshold. It is used to avoid a bias if the 3’ gene was not expressed and the 5’ gene was expressed at a very low level, i.e. low normal gene expression or not expressed chimaera (Table SX). The MDS was set to the C-score frequency maximum of a sample and considers the data quality of a sample; the higher the quality the lower the MDS. The colouring of a spot starts above or below the MDS, depending if there is a predicted absence or occurrence of the fusion transcript, otherwise the spot colour is set to white (Figure S6).

$R_{5´}=\frac{5´gene+1}{\overline{5´genes+1}} ; R_{3´}=\frac{3´gene+1}{\overline{3´genes+1}}$ (1)

$\begin{matrix} if R_{5´}\geq R_{3´} C\text{-}score=-R_{5´} \\ otherwise C\text{-}score=+R_{3´} \end{matrix}$ (2)

**Table S20.** Example data of ST read counts for *SLC45A3* and *ELK4* and a C-score without (eq 1 and 2) and without pseudo count of 1 (eq S1 and S2). The MDS is set to the maximum frequency of the C-score with pseudo count.

| Sample | | # Poly(A) tails of  5’ gene *SLC45A3* | | # Poly(A) tails of  3’ gene *ELK4* | | C-score  eqs 1 & 2 | C-score  pseudo count  eqs S1 & S2 | MDS |
| --- | --- | --- | --- | --- | --- | --- | --- | --- |
| Name | Spot coordinates | Spot value | Sample mean | Spot value | Sample mean |  |  |  |
| 2.3 | 25x12 | 3 | 6.5 | 0 | 0.3 | 0.0 | 0.8 | 0.8 |
| 2.3 | 22x4 | 4 | 6.5 | 0 | 0.3 | 0.0 | 0.8 | 0.8 |
| 3.2 | 11x17 | 3 | 12.8 | 0 | 1.0 | 0.0 | 0.5 | 0.5 |
| 3.2 | 12x22 | 4 | 12.8 | 0 | 1.0 | 0.0 | 0.5 | 0.5 |
| 3.2 | 11x25 | 5 | 12.8 | 0 | 1.0 | 0.0 | 0.5 | 0.5 |
| 4.2 | 21x23 | 3 | 8.5 | 0 | 1.1 | 0.0 | 0.5 | 0.5 |
| 4.3 | 9x13 | 3 | 7.9 | 0 | 0.8 | 0.0 | 0.55 | 0.55 |


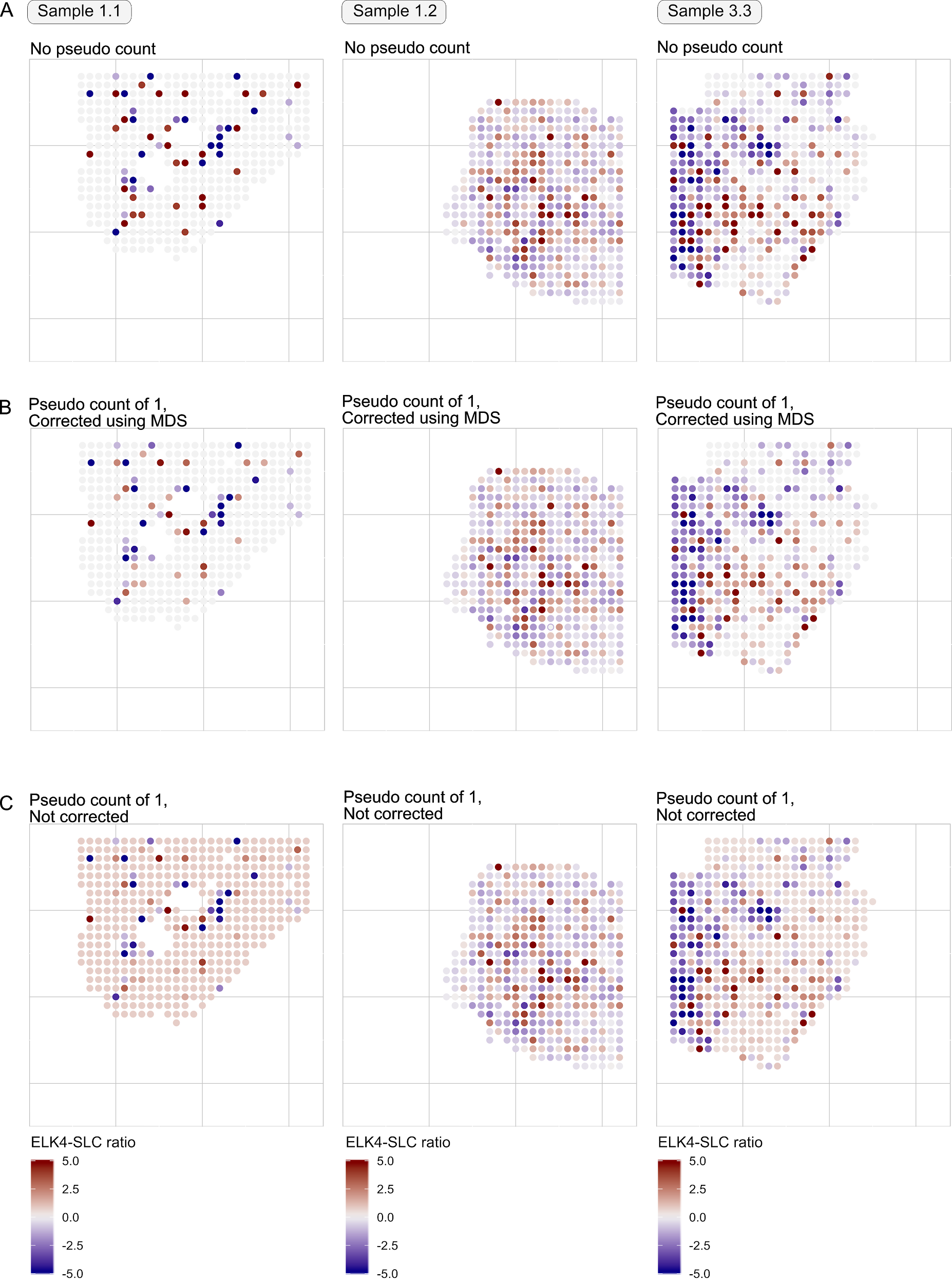


**Figure S6.** Comparison of C-score and C-score with pseudocount, the latter with and without correcting for MDS. There are only minimal differences in colour shading between the C-score without the pseudocount (A) and the C-score with pseudo count and correction using the MDS (B). Correction using the MDS as threshold corrects the bias (B and C). **A** C-score (eq 1 and 2). **B** C-score with pseudocount of 1 to avoid division of 0 (eq S1 and S2) and without correcting for bias using the minimal detectable signal MDS. **C** C-score with pseudo count of 1 (eq S1 and S2) but without correcting the bias using the minimal detectable signal MDS.

**Figure S7.** Distribution of C-scores with a pseudocount of 1. In each sample, except in sample 1.2 with very high ST data quality, a peak occurs between 0.35 and 0.8. The peak occurs if a C-score for a spot is calculated with a parental gene expression of 0 and a parental gene mean expression in a sample below 1. The minimal detectable signal (MDS) is set to the C-score frequency maximum. Red lines at the y-axis indicate the data points.


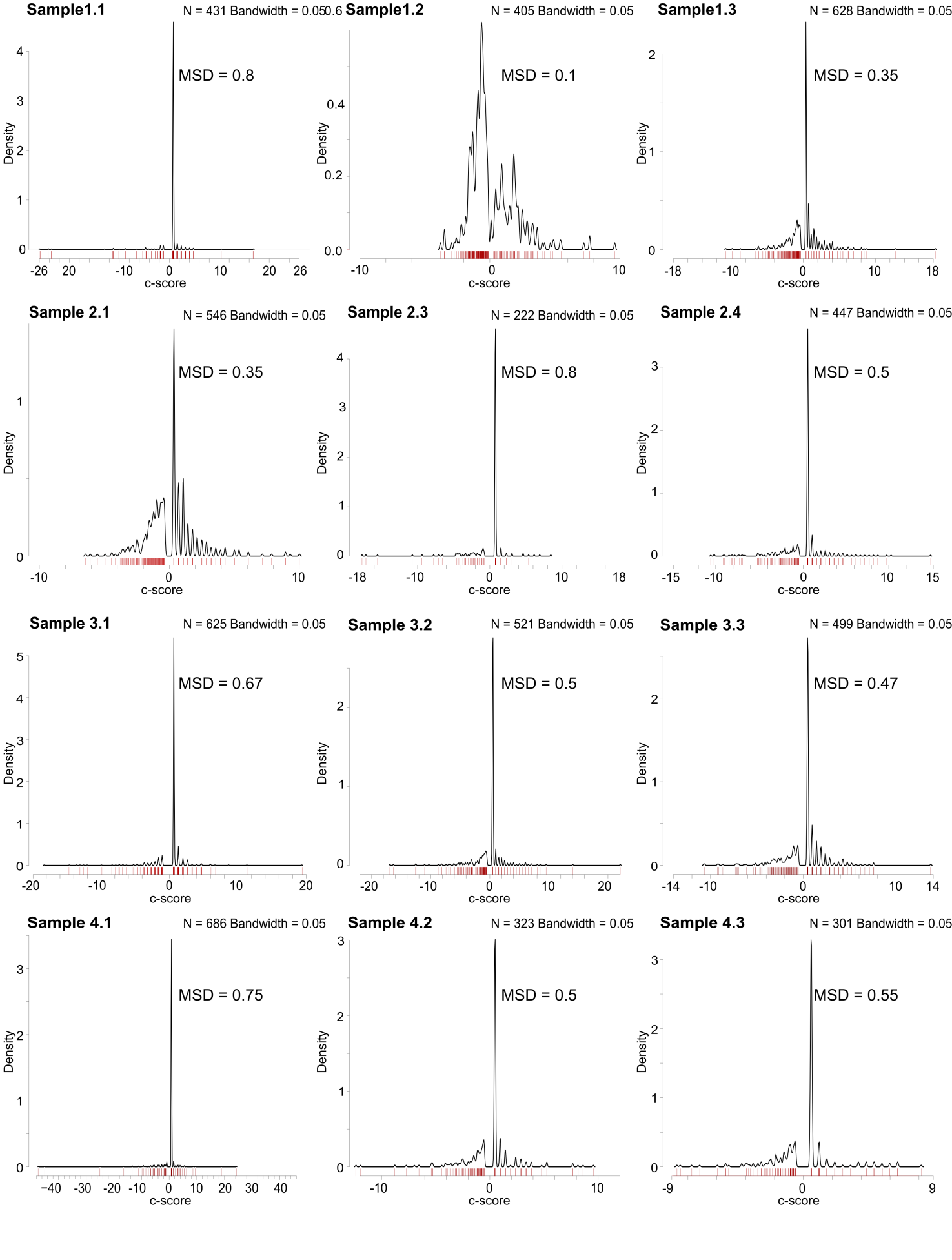

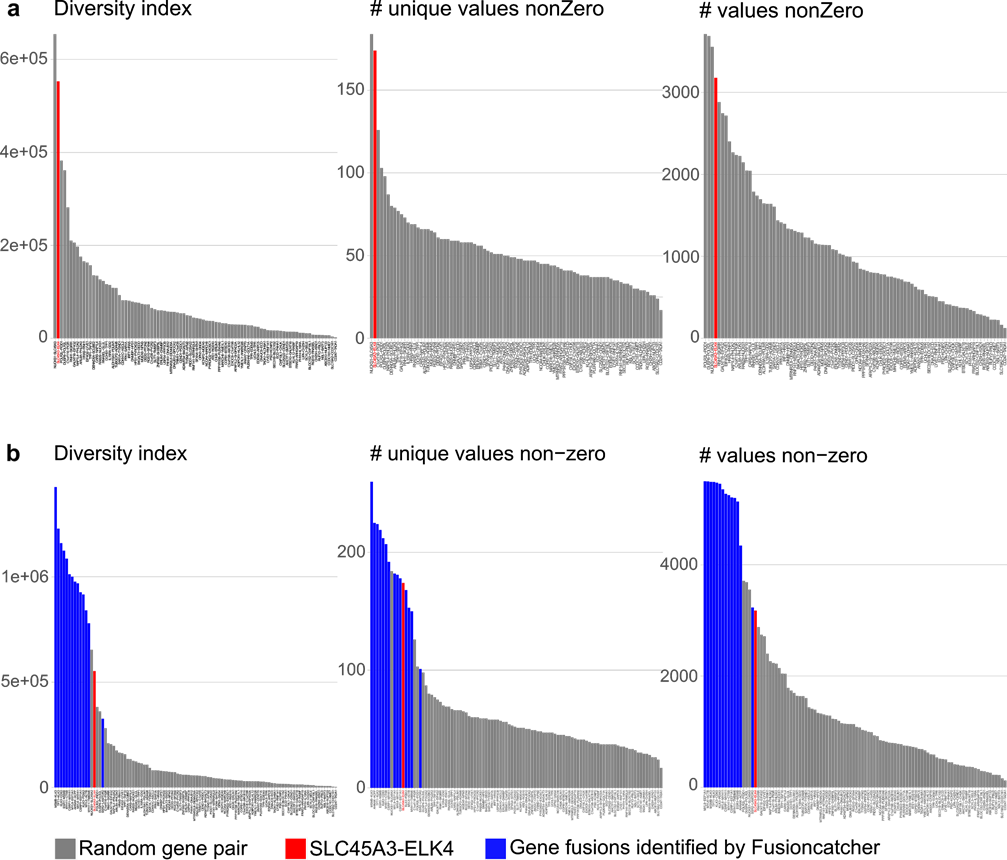


**Figure S8.** The diversity index helps to detect a novel fusion transcript. The diversity index is the multiplication of the number of unique non-zero C-scores and the number of non-zero C-scores. Shown are the benchmarks for the merged twelve clinical tissue samples to increase the amount of data. a. The benchmark was calculated for 100 random gene pairs having typical cis-SAGe characteristics described in Background (e.g. genetic neighbours, same strand) and the known cis-SAGe SLC45A3-ELK4. b. The gene fusions identified by Fusioncatcher in the twelve tissue samples were included in the plots (in blue).

## Non-canonical DNA-structures

A pre-mRNA, resulting from a failure in transcription initiation or termination, would contain all exons of both parental genes. However, in the studied samples, only fragments of the parental genes were observed, that is, not all exons of the parental genes were transcribed. This indicates that the transcription process neglects the corrupted sequence (Figure 1).

If the corrupted sequence starts or ends within an intron, the neighbouring exons are spliced and the fusion point is identified at the first or last base of these exons. However, if a fusion point was detected within an exon, the fusion point is in fact at this position. The fusion point of the 5´ gene of the cis-SAGe *SLC45A3* happens within exon 5 (Chr1:205,659,488).

**Table S21.** An i-Motif was found 7 bp downstream of the fusion point of the intra-exonic fusion point of the cis-SAGe *SLC45A3-ELK4* detected in the 12 clinical tissue samples. These non-canonical DNA structures are only a few nucleotides long (~10 nt) and regulate transcription by blocking [43].

| Chimeric transcript | Parental gene | Fusion point according to FusionCatcher | Strand | Motif sequence on genome | Non-canonical DNA structure |
| --- | --- | --- | --- | --- | --- |
| *SLC45A3- ELK4* | *SLC45A3* | Chr1:205,659,488 | reverse | CAC**CCACCACCA**CAC | i-Motif  (CCA)_3_ |
